# Anti-prostate cancer metabolites from the soil-derived *Aspergillus neoniveus*

**DOI:** 10.3389/fphar.2022.1006062

**Published:** 2022-10-14

**Authors:** Menna Fayek, Hassan Y. Ebrahim, Heba E. Elsayed, Mohamed S. Abdel-Aziz, Benson M. Kariuki, Fatma A. Moharram

**Affiliations:** ^1^ Department of Pharmacognosy, Faculty of Pharmacy, Helwan University, Helwan, Egypt; ^2^ Microbial Chemistry Department, Genetic Engineering and Biotechnology Division, National Research Centre, Giza, Egypt; ^3^ School of Chemistry, Cardiff University, Cardiff, United Kingdom

**Keywords:** prostate cancer, secondary metabolites, HPLC/MS, molecular modelling, x-ray

## Abstract

Prostate cancer (PCa) ranks as one of the most commonly diagnosed malignancies worldwide. Toxicity, lack of clinical efficacy, and development of resistance phenotypes are the main challenges in the control of prostate malignancies. Notably, castration-resistance prostate cancer (CRPCa) is a highly aggressive and metastatic phenotype of the disease with a poor prognosis and very limited therapeutic options. Herein, we report the isolation and genotypic identification of a soil-derived fungus *Aspergillus neoniveus* using the PCR-based internal transcribed spacer (ITS) region amplification approach. HPLC/MS investigation of the metabolic profile of the ethyl acetate extract from the fungal biomass revealed tentative identification of forty-five compounds belonging to various chemical classes including γ-butyrolactones, alkaloids, phenolics, and quinoids. Furthermore, the chromatographic purification of microbial extract enabled the identification of nervonic acid methyl ester (**1**) for the first time from endophytic fungi, as well as acetyl aszonalenin (**2**), and butyrolactone II (**3**) for the first time from *A. neoniveus*. The chemical frameworks of the isolated compounds were identified *via* extensive spectral analysis including 1 and 2D NMR and MS. The X-ray crystal structure and absolute configuration of acetyl aszonalenin (**2**) were also determined. Additionally, screening of *in vitro* anticancer activity of the fungal extract revealed its potential antiproliferative and anti-migratory activities against five different prostate cancer cells (PC3, PC-3M, DU-145, CWR-R1ca, and 22Rv1), including different cells with the castration-resistance phenotype. Moreover, the isolated metabolites significantly inhibited the proliferation, migration, and colonization of human prostate cancer cells at low micromolar levels, thus providing credence for future investigation of these metabolites in relevant anti-prostate cancer animal models. Furthermore, computational target prediction tools identified the cannabinoid G-protein coupled receptors type 1 (CB1) as a potential biological target mediating, at least in part, the anticancer effects of acetylaszonalenin (**2**). Moreover, molecular modeling and docking studies revealed a favorable binding pose at the CB1 receptor orthosteric ligand pocket aided by multiple polar and hydrophobic interactions with critical amino acids. In conclusion, the *Aspergillus neoniveus-*derived prenylated indole alkaloid acetylaszonalenin has promising anticancer activity and is amenable to further hit-to-lead optimization for the control of prostate malignancies *via* modulating CB1 receptors

## 1 Introduction

Prostate cancer (PCa) is one of the most commonly diagnosed malignancies, topping the cancer rankings in over half of the countries of the world in 2020 (Giona, 2021). According to recent statistics, the year 2020 witnessed the diagnosis of more than 1.4 million new prostate cancer cases and more than 375,000 victims of the disease consequences (Sung et al., 2021). Currently, there are various treatment options available for controlling PCa, including prostatectomy, cryotherapy, radiotherapy, hormonal therapy, chemotherapy, immunotherapy, and targeted therapy (Malik et al., 2019). Despite the initial success of different PCa management protocols, the development of resistant phenotypes worsen the disease prognosis, where limited therapies are available for resistant prostate malignant cells. For instance, after surgical prostatectomy, sometimes with castration, androgen-independent malignant cells may recur leading to dramatic failure to respond to standard androgen depletion therapy (ADT). This phenotype of the prostate malignancy is renowned as castration-resistant prostate cancer (CRPCa), which is very aggressive and highly metastatic. Furthermore, the modest clinical efficacy was shown by many prostate cancer therapeutics, including immune checkpoint inhibitors, vaccines, and targeted drugs also significantly limit the success of PCa management (Venkatachalam et al., 2021; Qi et al., 2022). Besides, the unavoidable side effects associated with current treatment options inflate the challenges towards the management of human PCa. For instance, urinary incontinence and erectile dysfunction are associated with radical prostatectomy, in addition to hair loss, nausea and vomiting, bleeding and anemia that frequently accompany the administration of chemotherapies (Beer and Bubalo, 2001; Kesch et al., 2021). Because of these challenges, research towards the discovery of new bioactive anti-prostate cancer hits and lead compounds is necessary for the development of novel and more tolerable clinical candidates for the control of human prostate malignancies.

Microbes, including fungi, are highly reputable for producing a plethora of chemically diverse metabolites which modulate a wide array of disease-relevant molecular targets uniquely and selectively. Since the discovery of penicillin, fungi have received attention directed at the exploration of their metabolic profiles aiming at identifying relevant biomolecules (Smedsgaard and Nielsen, 2005; Keller, 2019). For instance, the immunosuppressant ciclosporin, which has been isolated from the soil-derived ascomycetes fungus *Tolypocladium inflatum* is currently listed at the top of the World Health Organization’s list of essential medicines (World Heath Organization,). To date, although a substantial number of fungal-derived metabolites have reached the phases of the clinical trial, none of these candidates has yet been approved for clinical usage in cancer settings by any of the current drug authorities. Therefore, more research is still required to discover tolerable and effective anticancer therapies.

*Aspergillus neoniveus* is a fungal species of the genus *Aspergillus* and it belongs to the *Terrei* section (Gams et al., 1985). It was first reported by Samson and co-workers during the study of section *Terrei* using a polyphasic approach to characterize different isolates collected from soil samples that had been previously regarded as *A. terreus* (Samson et al., 2011). The approach included the partial gene sequence analysis of *β*-tubulin, calmodulin, and ITS region, and the examination of extrapolating profiles. Notably, the *Aspergillus* species of the section *Terrei* are renowned for the biosynthesis of diverse secondary metabolites including terpenoids, polyketides, *β*-butyrolactones, alkaloids, xanthones, anthraquinones, and steroids. Potential biological activities have also been linked to these metabolites, including cholesterol-lowering, anti-inflammatory, antioxidant, antimicrobial, antiviral, antiparasitic, immunosuppressive, antiangiogenic, and anticancer activities. Nevertheless, very little is known regarding the chemical and biological profiles of *A. neoniveus*.

Understanding the molecular mode of action is a fundamental step in the early stages of anticancer drug discovery. The phenotypic screening assays have shown robust outcomes in the identification of bioactive anticancer hits from chemical libraries or complex extracts of natural products. Afterward, comprehensive biochemical and pharmacological studies must be set up to uncover relevant macromolecular targets mediating the observed biological response. Experimental target identification can be attained utilizing various approaches such as proteomic analyses and affinity chromatography, but they are usually time-consuming and cost expensive. Conversely, computational-based approaches can swiftly predict primary molecular targets of bioactive hits identified from phenotypic screening assays, and even at next to no cost in many cases. In principle, there are three main procedures to attain experimental target hitting, including ligand-based, structure-based, and hybrid approaches (Sydow et al., 2019). In particular, the ligand-based strategy utilizes structure similarity in the search methodology, based on the notion that similar structures possess a similar biological activity. Therefore, computational target identification is currently getting more attention leading to routine applications in the drug discovery pipeline.

Accumulated evidence supports the significant role of cannabinoid receptors in prostate cancer progression and metastasis. For instance, treatment of LNCaP prostate cancer cells with a cannabinoid receptors agonist WIN 55,212–2 inhibited the neuroendocrine differentiation through downregulation of the PI3K/AKT/mTOR pathway (Morell et al., 2016). Furthermore, treatment of animals xenografted with PC-3 and DU-145 human prostate cancer cells by WIN 55,212–2 significantly hampered tumor growth in a dose-dependent manner (Roberto et al., 2019). At the molecular level, cannabinoid receptors belong to class A G-protein coupled receptors (GPCRs), with two types being known: CB1 and CB2. CB1 is the most abundant GPCR in the CNS and mediates diverse cellular functions. Cannabinoid receptors have the classical seven-transmembrane fold (TM I-VII) of GPCRs with the *C*-terminal directed intracellular and *N*-terminal directed extracellular (Shao, et al., 2016). The ligand orthosteric binding pocket is located in the space between helices III and VII of the extracellular side. Moreover, the ligand binding cavity is lined with various hydrophobic amino acids to accommodate lipophilic endocannabinoids (arachidonoyl glycerol and *N*-arachidonoyl ethanolamine). Therefore, modulating cannabinoid receptors with lipophilic small molecules would be a promising strategy to control malignancies of the prostate.

Herein we report the isolation of *A. neoniveus* from a soil sample, as well as tentative identification of the ethyl acetate metabolites using HPLC/MS and isolation of metabolites from it. The structure and absolute configuration of acetylaszonalenin (**2**) were confirmed by single crystal X-ray crystallography. In addition, screening for *in vitro* anticancer activity of the extract and isolated metabolites against five prostate cancer cells is reported. Furthermore, computational target prediction tools were used to establish the anticancer mechanism, at least in part, associated with the anticancer effects of acetylaszonalenin.

## 2 Materials and methods

### 2.1 General

Silica gel 60 for column chromatography (CC) and pre-coated silica gel 60 F_254_ plates for thin layer chromatography (TLC) were obtained from Merck (Darmstadt, Germany). ^1^H and ^13^C NMR experiments were recorded on a Bruker Avance spectrometer (Bruker, Rheinstetten, Germany) at 400 and 100 MHz, respectively. Spectral data were represented as *δ* in ppm relative to TMS as an internal reference. HPLC/MS and ESI-MS analyses were carried out on a XEVO TQD triple quadrupole LC/MS/MS. (Waters Corporation, Milford, MA01757 U.S.A). Chromatographic conditions; column: ACQUITY UPLC—BEH C18 1.7 µm—2.1 × 50 mm, flow rate: 0.2 ml\min; solvent system: water containing 0.1% formic acid and acetonitrile containing 0.1% formic acid. All solvents used in the isolation of compounds were of analytical grade and supplied from El Nasr Pharmaceutical Chemicals Company (Cairo, Egypt).

### 2.2 Fungal material

Soil samples were collected from an agriculture field at 10 cm depth in Dekernis City, Dakahlia Governate, Egypt in December 2019. Samples were sieved and air dried at room temperature for 3–5 days and kept at 4°C till the investigation. A soil suspension was prepared by suspending 20 g of the collected soil in 200 ml sterilized distilled water, shaken for 3 h on a rotary shaker, and then left to settle for 30 min. For the isolation of fungal strains, about 500 µL of the supernatant was transferred into a sterile slant containing 5 ml sterilized water and then used to prepare samples by serial dilution 10^−1^ to 10^−5^. Each dilution was inoculated in potato dextrose agar (PDA) plates (100 ml; 200 g potato, 20 g glucose, and 15 g agar in purified water (Innis et al., 2012) supplemented with neomycin (125 mg/L) for suppressing the bacterial growth. The plates were incubated at 30 ± 2°C for 6–8 days, and growth was observed after 2 days. Growing colonies with different morphological appearances were selected and transferred onto fresh PDA media and kept at 40°C. Afterward, different pure strains were isolated by repeated and successive sub-culturing.

### 2.3 Identification of the isolated fungal strain

Fungal strain identification was attained by molecular biological protocol through DNA amplification and sequencing of the internal transcribed spacer (ITS) region which represents the most effective, fast, and easy technique (Alwakeel, 2013). A fungal culture piece was suspended in sterilized saline (0.5 µL) and then centrifuged at 10.000 rpm for 10 min and the pellet remaining after removal of the supernatant was suspended in 0.5 µL of Insta Gene Matrix kits (Bio-Rad, United States) and incubated for 30 min at 56°C followed by heating for 10 min at 100°C. Polymerase chain reaction (PCR) was used for amplification of the nuclear ribosomal internal transcribed spacers (ITS rDNA) as single fragments of StarTaq Master Mix Kit and DNA template (Bio-Rad, Hercules, Ca) were mixed with pair of primers ITS1 (5'-TCC GTA GGT GAA CCT GCG G-3') and ITS4 (5'-TCC TCC GCT TAT TGA TAT GC-3') in an I Cycler. The thermal cycle was carried out as follows: the first holding step was done for 15 min at 95°C to activate the Hot StarTaq® DNA polymerase; the second denaturation step for 1 min at 95°C, and the third was annealing for 0.5 min at 56°C, then the extenuation fourth step at 72°C for 1 min followed by the final extenuation step at 72°C for 10 min. Steps 2- 4 were repeated 35 times (Liao et al., 2012). For PCR product purification, 20 µL was loaded onto agarose gel (2% agarose in TBA buffer, 5 µL of ethidium bromide 1% m/V solution/100 ml of gel) followed by electrophoresis at 70 V for 60 min. The UV fluorescent PCR product band was isolated from the gel slice using the Gen Elute™ Gel extraction kit following the manufacturer’s protocol. The corresponding fungus ITS-rDNA sequence was used for similarity investigation *via* the Blast N algorithm against the public database at the National Centre for Biotechnology Information (NCBI;http://www.ncbi.nlm.nih.gov). The pooled results and the phylogenetic tree were constructed by Molecular Evolutionary Genetics Analysis (MEGA) version 10.0.5 (https://www.megasoftware.net/). A voucher strain was reserved at Microbial Chemistry Department, National Research Centre, Dokki, Giza, Egypt.

### 2.4 Cultivation of *Aspergillus neoniveus* for production of secondary metabolites

The scale-up of the pure cultivated fungal biomass growth was accomplished by transferring pieces from PDA media to Erlenmeyer flasks (10 × 1L) containing solid rice as solid culture media (100 g rice and 150 ml sterile water). Finally, the fungus was incubated at 30°C for 14 days under static conditions and checked periodically for fungal growth.

### 2.5 HPLC/ESI-MS for ethyl acetate extract of *Aspergillus neoniveus*

Different metabolites of the ethyl acetate extract were tentatively identified using HPLC/ESI-MS in both positive and negative ionization modes. The identified compounds are listed in Table 1, and their chemical structures are shown in Supplementary Figures S1–S3.

**TABLE 1:** Identified compounds from ethyl acetate extract of *A. neoniveus* by HPLC/ESI-MS.

Rt (min)	MS ion peak		Molecular Formula	Potential metabolite(s)	Reference
	[M-H]^ˉ^	[M+H]^+^			
1.05		229.1290	C_14_H_12_O_3_	Reversetrol **(1)**	Abdel-Wareth and Ghareeb (2018)
1.06	205.1757		C_12_H_14_O_3_	Terreprenphenol C **(2)**	Li et al. (2019)
				4-Hydroxy-3-prenyl benzoic acid **(3)**	
				Asperterreusine C **(4)**	
5.85	229.2755		C_10_H_18_N_2_O_4_	Terramide C **(5)**	Garson et al. (1986)
6.08		245.1974	C_14_H_16_N_2_O_2_	Cyclo (phenyl alanine-proline) **(6)**	Xiao et al. (2019)
			C_11_H_20_N_2_O_4_	Terramide B **(7)**	Garson et al. (1986)
6.69		223.1978	C_12_H_14_O_4_	(3R)-3,4-dihydro-6,8-dimethoxy-3-methylisocoumarin **(8)**	Sun et al. (2018)
				Terreprenphenol B **(9)**	Li et al. (2019)
				Anodendroic acid **(10)**	
7.59		249.1496	C_15_H_20_O_3_	Quadrone **(11)**	Wijeratne et al. (2003)
				(‒)-Isoquadrone **(12)**	
				Terrecyclic acid A **(13)**	
	265.2383		C_13_H_14_O_6_	Dihydrocitrinone **(14)**	El‐Hawary et al. (2021)
			C_17_H_14_O_3_	Xenofuranone B **(15)**	Parvatkar et al. (2009)
			C_15_H_22_O_4_	Aspterric acid **(16)**	Li et al. (2020)
				(+)-5(6)-dihydro-6-hydroxyterrecyclic acid A **(17)**	Wijeratne et al. (2003)
8.24		399.2597	C_23_H_30_N_2_O_4_	Fumigaclavine I **(18)**	Li et al. (2015)
			C_28_H_46_O	Ergost-4-ene-3-one **(19)**	Hamed et al. (2020)
			C_23_H_26_O_6_	(−)-Asperteretone A **(20)**	Liu et al. (2018)
				(+)-Asperteretone A **(21)**	
				Asperteretal G1 **(22)**	Yang et al. (2018)
				Asperteretal G2 **(23)**	
				Asperteretal H **(24)**	
			C_24_H_18_N_2_O_4_	Asterriquinone D **(25)**	Girich et al. (2020)
8.25	355.2412	357.2113	C_19_H_16_O_7_	Butyrolactone II **(26)**	Yang et al. (2018)
8.77		251.1632	C_13_H_14_O_5_	Aspergiketal **(27)**	El‐Hawary et al. (2021)
				Terreinol **(28)**	Chen et al. (2015)
	307.2572		C_18_H_12_O_5_	Pulvic acid **(29)**	Gao et al. (2013)
9.50		478.4089	C_27_H_31_N_3_O_5_	Varioxepine B **(30)**	Qi et al. (2020)
9.81	431.3432		C_27_H_28_O_5_	Aspulvinone B **(31)**	Ojima et al. (1975)
				Aspulvinone H **(32)**	Chen et al. (2018)
10.08		381.2989	C_23_H_24_O_5_	(−)-Asperteretone B **(33)**	Liu et al. (2018)
				(+)-Asperteretone B **(34)**	
				(−)-Asperteretone D **(35)**	
				(+)-Asperteretone D **(36)**	
				(±)-Asperteretal D **(37)**	
			C_22_H_20_O_6_	Asperjinone **(38)**	Liao et al. (2012)
			C_23_H_24_O_5_	(4S)-4-decarboxyl flavipesolide C **(39)**	Liu et al. (2018)
10.88		416.2879	C_25_H_25_N_3_O_3_	Epi-aszonalenin A **(40)**	Chaiyosang et al. (2016)
11.06	279.2821		C_18_H_16_O_3_	Xenofuranone A **(41)**	Parvatkar et al. (2009)
			C_18_H_32_O_2_	Linoleic acid **(42)**	Shaaban et al. (2018)
			C_16_H_24_O_4_	(+)-5(6)-dihydro-6- methoxyterrecyclic acid A **(43)**	Wijeratne et al. (2003)
22.21		284.4075	C_16_H_17_N_3_O_2_	Brevianamide F **(44)**	Xiao et al. (2019)
24.62		413.3689	C_29_H_48_O	β-Sitostenone **(45)**	Shaaban et al. (2018)

### 2.6 Isolation of the main secondary metabolites

The rice culture media were extracted three times till exhaustion with ethyl acetate (250 ml). The remaining marc was re-extracted with 250 ml of the acetone-water mixture (50:50, v/v) and the combined extracts were filtered, and dried under vacuum affording approximately 8.0 g sticky mass. About 6.0 g of the extract were adsorbed on 50 g of silica gel and fractionated using a vacuum liquid chromatography (VLC) approach using silica gel 60 (500 g, 12 × 20 cm), and starting the elution with dichloromethane and then gradually increasing the polarity by MeOH (5%, 10%, 20% till 100% MeOH). Five collective fractions were pooled up based on their chromatographic behavior on TLC using UV-light, followed by spraying with *p*-anisaldehyde/sulfuric acid and heating at 110°C till full-color development. Fraction I (100% DCM, 1.0 g) was fractionated on silica gel CC using *n*-hexane/dichloromethane (70:30 v/v) as an eluent to afford pure compound **1** (100 mg). Fraction II (dichloromethane: MeOH, 90:10 v/v, 3.0 g) was fractionated on silica gel CC with isocratic elution by dichloromethane: MeOH (94:4 v/v) to afford two subfractions A and B. Fraction IIA (225 mg) was purified on repeated silica gel CC using *n*-hexane/ethyl acetate (60:40 v/v) as mobile phase to afford the chromatographically pure compound **2 (**15 mg**)**. The second subfraction (IIB, 220 mg) was chromatographed on silica gel CC and eluted with DCM/ethyl acetate (60:40 v/v) to afford compound **3** (13 mg). The purity of the isolated compounds was checked by TLC and HPLC analysis.

Compound **1** was obtained as a yellow oil and gave a violet color upon spraying and heating with *p*-anisaldehyde/sulfuric acid. ^1^HNMR and ^13^CNMR were recorded in CDCl_3_ at 400 and 100 MHz respectively. Positive ESI-MS showed a quasi-molecular ion peak at *m/z* 381.2989 [M + H]^+^. The 1 and 2D NMR and ESI-MS spectra are provided in Table 2 and Supplementary Figures S4–S10.

**TABLE 2:** ^1^HNMR and ^13^CNMR data of compound 1 (CDCl_3_, 400 MHz and 100 MHz respectively).

C	δ_C_ (ppm)	δ_H_ (ppm)	^1^H–^1^H COSY	HMBC
**1**	173.9 C	-		
**2**	33.9 CH_2_	2.27 (t, 7.6)	H-3	C-1, 3, 4
**3**	24.9 CH_2_	1.59 (m)	H-4, 2	C-1, 5
**4**	31.9 CH_2_	1.28 (m)	H-3	C-6
**5**	29.2 CH_2_	1.28 (m)		
**6**	29.3 CH_2_	1.28 (m)		
**7**	29.3 CH_2_	1.28 (m)		
**8**	29.4 CH_2_	1.28 (m)		
**9**	29.5 CH_2_	1.28 (m)		
**10**	29.5 CH_2_	1.28 (m)		C-12
**11**	29.6 CH_2_	1.28 (m)		
**12**	29.6 CH_2_	1.28 (m)		
**13**	29.7 CH_2_	1.28 (m)	H-14	
**14**	27.1 CH_2_	1.98 (m)	H-15, 13	C-12, 15
**15**	129.9 CH	5.31 (m)	H-14	C-14
**16**	129.6 CH	5.31 (m)	H-17	C-17
**17**	27.1 CH_2_	1.98 (m)	H-16, 18	C-15, C-18
**18**	29.0 CH_2_	1.28 (m)	H-17, 19	
**19**	24.9 CH_2_	1.59 (m)	H-20, 18	
**20**	29.1 CH_2_	1.28 (m)	H-19	
**21**	29.1 CH_2_	1.28 (m)		
**22**	31.9 CH_2_	1.28 (m)		C-23
**23**	22.6 CH_2_	1.28 (m)		
**24**	13.9 CH_3_	0.86 (t-like, 7.2, 6.4)		C-22, 23
**1′**	51.2 CH_3_	3.63 (s)		C-1

Value between parenthesis represent the J value in Hz.

Compound **2** was isolated as white crystals and displayed pink color upon spraying and heating with *p*-anisaldehyde/sulfuric acid. The negative ESI-MS showed a quasi-molecular ion peak at 414.3071[M-H]ˉ. The 1 and 2D NMR and ESI-MS spectra are provided in Supplementary Figures S11–S17 and the chemical shifts, splitting patterns, and correlations are listed in Table 3.

**TABLE 3:** ^1^H and ^13^C-NMR data of compound 2 (CD_3_OD, 400 MHz and 100 MHz, respectively).

C	δ_C_ (ppm)	δ_H_ (ppm)	^1^H-^1^H COSY	HMBC
**2**	82.1 (CH)	5.94 (s)	---	C-8, 9, 3ˊ, 4ˊ
**3**	60.2 (C)	---	---	---
**4**	119.2 (CH)	8.01 (m)	H-6	C-3´, 9
**5**	124.0 (CH)	7.08 (m)		C-4, 6, 9
**6**	128.9 (CH)	7.29 (m)	H-4	C-7, 8
**7**	124.3 (CH)	7.26 (dd, 7.6, 2.4)	---	C-6, 8
**8**	142.0 (C)	---	---	---
**9**	133.8 (C)	---	---	---
**10**	30.6 (CH_2_)	2.44 (dd, 13.6, 8.4; H10a)	H-10b, 11	C-3, 9, 2
	---	3.39 (dd, 13.6, 8.4; H10b)	H-10a, 11	C-3ˊ, 11, 3, 9, 17
**11**	56.6 (CH)	3.91 (dd, 8.4, 8.0)	H-10a, H-10b	C-10
**13**	166.6 (C)	---	---	---
**14**	127.5 (C)	---	---	---
**15**	133.8 (C)	---	---	---
**17**	169.6 (C)	---	---	---
**18**	125.4 (CH)	7.15 (m)	H-20	C-15, 14, 21
**19**	132.3 (CH)	7.39 (ddd, 8.4, 8.4, 1.2)	H-21	C-20, 15, 18
**20**	131.0 (CH)	7.67 (dd, 7.6, 0.8)	H-18	C-13, 15
**21**	120.6 (CH)	6.96 (d, 8.0)	H-19	C-18, 13
**1ˊ**	114.2 (CH_2_)	5.10 (ddd, 14.4, 11.6, 2.8; H1ˊa)	---	C-3ˊ
		5.13 (br.d; H1ˊb)	H-2ˊ	C-3ˊ
**2ˊ**	143.3 (CH)	5.89 (dd, 17.6, 10.4)	H-1ˊb	C-3ˊ, 5ˊ
**3ˊ**	40.7 (C)	---	---	---
**4ˊ**	22.5 (CH_3_)	1.19 (s)	---	C-3, 2ˊ, 3ˊ, 5ˊ
**5ˊ**	22.9 (CH_3_)	0.99 (s)	---	C-3, 2ˊ, 3ˊ, 4ˊ
**COCH**_**3**_	24.1 (CH_3_)	2.61 (s)	---	COCH_3_
**COCH**_**3**_	170.6 (C)	---	---	---

Value between parenthesis represent the J value in Hz.

Compound **3** was obtained as yellow amorphous powder and displayed a pink color upon spraying and heating with an anisaldehyde/sulfuric acid reagent. Negative ESI/MS showed a quasi-molecular ion peak at *m/z*. 355.2861 [M- H]^-^ (calc for C_19_H_16_O_7_). The 1 and 2D NMR and ESI-MS spectra are provided in Table 4 and Supplementary Figures S18–S24.

**TABLE 4:** ^1^H and ^13^C-NMR data (CD_3_OD, 400 MHz, and 100 MHz respectively) for compound 3 and comparison with reported literature.

C No	δ C (ppm)	δ H (ppm)			^1^H-^1^H COSY	HMBC
	3	Reported literature*	3	Reported literature*		
**1**	169.3	168.4				
**2**	138.5	138.5				
**3**	127.9	127.9				
**4**	85.6	85.1				
**5**	38.1	38.4	3.49 (m)	3.4 (dd, 14.7, 8.1)		C-3, 4, 6, 1ˊˊ, 2ˊˊ, 6´´
**6**	170.2	170.2				
**7**	52.7	53.9	3.78 (s, 3H)	3.74 (s)		C-6
**1´**	121.8	121.4				
**2´/6´**	129.1	129.2	7.62 (d, 8.4)	7.52 (d, 8.6)	H-3ˊ, 5ˊ	C-3, 4ˊ, **3´/5**, 4ˊ
**3´/5´**	115.4	116.3	6.90 (d, 8.4)	6.59 (d, 8.6)	H-2ˊ, 6ˊ	C-1ˊ, 4ˊ
**4´**	157.8	158.3				
**1´´**	123.9	123.6				
**2´´/6´´**	131.3	131.6	6.67 (d, 8.4)	6.88 (d, 8.8)	H-3ˊˊ, 5ˊˊ	3ˊˊ/5ˊˊ, 4ˊˊ, C-5
**3´´/5´´**	114.3	115.1	6.55 (d, 8.4)	6.51 (d, 8.4)	H-2ˊˊ, 6ˊˊ	C-1ˊˊ, 2ˊˊ/6ˊˊ, 4ˊˊ
**4´´**	155.9	156.7				

Value between parenthesis represent the J value in Hz.

*Yang et al., 2018 (600 and 125 for ^1^H and ^13^CNMR, DMSO-d_6_).

### 2.7 Crystal structure determination

Single-crystal XRD data were collected at 23°C on an Agilent SuperNova Dual Atlas diffractometer with a mirror monochromator using Cu radiation. The crystal structure was solved by SHELXS (Sheldrick, 2008) and refined using SHELXL (Sheldrick, 2015). Non-hydrogen atoms were refined with anisotropic displacement parameters. Hydrogen atoms were inserted in idealized positions, and a riding model was used with Uiso set at 1.2 or 1.5 times the value of Ueq for the atom to which they are bonded. Crystal and refinement data are shown in Table 5. The crystal structure has been deposited in the Cambridge Structural Database under reference CCDC 2189133.

**TABLE 5:** Crystal data and structure refinement for compound 2.


Formula	C_25_H_25_N_3_O_3_, H_2_O	Absorption coefficient (mm^−1^)	0.726
Formula weight	433.49	F (000)	1380
Temperature (K)	296(2)	Crystal size (mm^3^)	0.359 × 0.149 × 0.092
Wavelength (Å)	1.54184	Reflections collected	24883
Crystal system	Hexagonal	Independent reflections	4228
Space group	P 6_1_	R(int)	0.0310]
a (Å)	21.4572(3)	Parameters	315
b (Å)	21.4572(3)	Goodness-of-fit on F^2^	1.057
c (Å)	8.29210(10)	R1 (I>2σ(I))	0.0326
a (°)	90	wR2 (I>2σ(I))	0.0852
b (°)	90	R1 (all data)	0.0357
g (°)	120	wR2 (all data)	0.0882
Volume (Å^3^)	3306.29(10)	Absolute structure parameter	0.00(9)
Z	6	Extinction coefficient	0.0022(3)
Calculated Density (Mg/m^3^)	1.306	Largest diff. peak and hole	0.108 and -0.117 e.Å^−3^

### 2.8 Anticancer activity

#### 2.8.1. Cell lines and culture conditions

The *Homo sapiens* prostate carcinoma epithelial cell lines PC-3, PC-3M, DU-145, 22Rv1 were obtained from the American Type Culture Collection (ATCC, Manassas, VA, United States). CWR-R1ca cells were kindly provided by Dr. Elmageed, Edward *via* College of Osteopathic Medicine, Monroe, Louisiana, United States. All prostate cancer cells were cultured in (RPMI-1640) (Corning, Manassas, VA, United States) supplemented with 10% fetal calf serum (R&D Systems, Inc., Minneapolis, MN, United States) and 1% antibiotic/antimycotic solution (Corning, Manassas, VA, United States). Cells were maintained in a humidified incubator (VWR, Radnor, PA, United States) at 37°C and 5% CO_2_ and checked periodically for confluency. When the cells’ monolayers were about 80–90% confluent, cells were washed with phosphate-buffered saline (PBS) and detached by 0.05% trypsin/EDTA (Corning, Manassas, VA, United States) for 5–10 mins at 37°C and 5% CO_2_ for subsequent culturing.

#### 2.8.2 Cell proliferation assay

Cells were suspended in RPMI-1640 medium and seeded into a 96-well plate at a cell density 1 × 10^4^ cells/well and then left overnight to adhere to the base of the well. Then, seeding media were aspirated and cells were washed with PBS and treated with 100 µL of RPMI-1640 supplemented with 1% fetal calf serum containing different concentrations of either the microbial extract (10 mg/ml DMSO stock) or the purified compounds (10 mM DMSO stock) or DMSO (negative control) in triplicate and cells were left in the incubator for 48 h. At the end of the incubation period, media were removed, and cells were washed with PBS. About 100 µL of RPMI-1640 containing 3-(4,5-dimethylthiazolyl2)-2,5-diphenyltetrazolium bromide (MTT) at a concentration of 0.5 mg/ml were added to each well, and the cells were further incubated for 4 h at 37°C till the formation of insoluble formazan crystals. Then, media were removed, and 100 µL DMSO was added to each well to solubilize the formazan crystals. Finally, the absorbance was measured at 570 nm using a microplate reader (BioTek, Winooski, VT, United States).

#### 2.8.3 Cell motility assay

Cells were seeded into a 24-well plate in a cell density of 2 × 10^5^ cells/well and incubated overnight to adhere. The next day, scratches were created in the cell monolayer using a sterilized 200 µL pipette tip. Media were then removed, and the cells were washed two times with PBS to remove detached cells and debris. The treatment concentrations of either the extract (10 mg/ml DMSO stock) or the purified compounds (10 mM DMSO stock) were prepared in RPMI-1640 (Corning, Manassas, VA, United States) supplemented with 1% fetal calf serum (R&D Systems, Inc., Minneapolis, MN, United States). About 500 µL of each treatment concentration were added to each well (in triplicate) and only DMSO-containing media as vehicle control. Cells were then incubated at 37°C and 5% CO_2_ in a humidified atmosphere and monitored periodically for wound closure. Upon wound closure, media were gently removed, and cells were washed two times with cold PBS and fixed with cold methanol on ice for 15 min. Then, alcohol was removed, and plates were allowed to dry at rt, followed by staining with 200 µL/well Giemsa stain solution (Sigma Aldrich, St. Louis, MO, United States) for 15 min before gently washing under tap water. Images were captured immediately at the time of wound creation (*zero time*) and the end of the incubation period for each treatment concentration. Images were then used to calculate percent cell migration relative to DMSO-treated control cells as follows:

Percent cell proliferation $=\left[1-\left(\frac{Wt-Wcont}{W0-Wcont}\right)\right]x\;100$

*W*_*t*_: wound at the end of the experiment in treatment wells; *W*_*0*_: wound at the time of wound creation (*zero time*); *W*_*cont*_: wound at the end of the experiment in control wells.

#### 2.8.4 Clonogenic cell survival assay

About 500 cells/well were seeded in a 12-well plate in 1 ml RPMI-1640 medium supplemented with 10% fetal calf serum and left to adhere for 3 days in a humidified incubator at 37°C and 5% CO_2_. Next, media were gently removed, and cells were washed with PBS. Cells were then treated with a test compound (10 mM DMSO stock) at indicated concentrations in RPMI-1640 (Corning, Manassas, VA, United States) supplemented with 10% fetal calf serum (R&D Systems, Inc., Minneapolis, MN, United States). Treatment media were changed every 72 h with test compound or DMSO in vehicle-control treated cells. Cells were monitored for the formation of distinguished colonies for 15–21 days. At the end of incubation periods, media were removed, and cells were washed with PBS and fixed with ice-cold methanol for 15 min Then, alcohol was aspirated, and wells were allowed to dry before adding Giemsa stain solution (Sigma Aldrich, St. Louis, MO, United States) for visualization. After staining, wells were photographed, and colonies were counted and normalized to vehicle-treated control cells (representing 100% colonization).

### 2.9 Computational molecular target prediction

The Swiss Target Prediction (available at http://www.swisstargetprediction.ch/) was implemented to search for potential macromolecular targets that could mediate the anticancer effects of acetylaszonalenin (**2**). Firstly, the chemical structure of **2** was sketched on the ChemDraw Professional molecular interface (version 15.0, PerkinElmer Informatics, Waltham, MA, United States), converted to MDL Mol file, and imported at Marvin JS online interface. *Homo sapiens* was selected as relevant species for computational target search.

### 2.10 Molecular modeling and docking studies

The *in-silico* computational experiments were accomplished utilizing the Chemical Computing Group’s Molecular Operating Environment (MOE) 2014.09 release. The software package was installed on a SAMSUNG laptop computer with Intel(R) Core (TM) i7-6500U CPU at 2.5 GHz processor and 12.0 GB RAM.

#### 2.10.1 Receptor structure preparation

The X-ray crystal structure of the human membrane protein cannabinoid receptor type 1 (CB1, PDB ID: 5xR8) was retrieved from the Protein Data Bank (available at https://www.rcsb.org/). The structure preparation module of MOE was used to optimize the 3D receptor, where structural issues such as incorrect charges, alternates, termini, and hydrogen count were addressed and resolved. The protonate3D module of the MOE was used to identify residues with possible rotamers, protomers, or tautomeric states. Finally, energy minimization was executed using MMFF94x forcefield at default parameters.

#### 2.10.2 Ligand structure preparation

The 2D structure of acetylaszonalenin (**2**) was sketched using ChemDraw Professional molecular interface (version 15.0, PerkinElmer Informatics, Waltham, MA, United States) and saved as an MDL Mol file. Acetylaszonalenin was then imported at the MOE interface, and the 3D structure was generated for conformational search. The geometry optimization and energy minimization were utilized for generating energetically stable 3D conformers.

#### 2.10.3 Molecular docking

The X-ray crystal structure of the human membrane protein cannabinoid receptor type 1 (CB1, PDB: 5xR8) and the 3D energetically minimized conformer of acetylaszonalenin was implemented for the docking experiments. The Rigid Receptor docking protocol was set for all docking studies, using the Triangle Matcher for Placement and the London dG for Rescoring and Force Field for Refinement for the final.

### 2.11 Statistical analysis

All biological experiments were conducted in triplicates at least three times. Data analysis was accomplished using the GraphPad Prism Software version 5.0 (La Jolla, CA, United States). Reported data represent the calculated mean ± SD. Means were compared using the student *t*-test, where *p* value <0.05 is considered statistical significance relative to vehicle-control treated cells.

## 3 Results and discussion

### 3.1 Molecular identification of the *Aspergillus neoniveus*

Fungi are the largest group of eukaryotes with the estimated number of species exceeding five million (Iquebal et al., 2021). The identification of fungal species is a paramount milestone in numerous fields including taxonomy, ecology, agriculture, industry, as well as health sciences. Morphological, biochemical, and molecular approaches are well-established techniques for fungal identification. The amplification of the DNA barcode of the internal transcribed spacer (ITS) is considered the gold standard in the molecular identification of fungal species. Herein, the collected soil sample, which was processed as stated earlier in the experimental section, afforded a filamentous fungus with yellowish conidiophores and conidia. The fungal genetic material was successfully extracted using the Insta Gene Matrix kit followed by PCR analysis. Results from similarity search revealed that the fungus 18S ribosomal RNA sequence exhibited 100% similarity with *Aspergillus neoniveus*. Data were deposited at the GenBank (https://www.ncbi.nlm.nih.gov/genbank/) under the accession number MW035695. The phylogenic tree of *A. neoniveus* showing its relationship with other species based on their sequence homologies of 18S rRNA is depicted in Supplementary Figure S25.

### 3.2 HPLC/ESI-MS analysis and isolation of main metabolites

**FIGURE 1:**
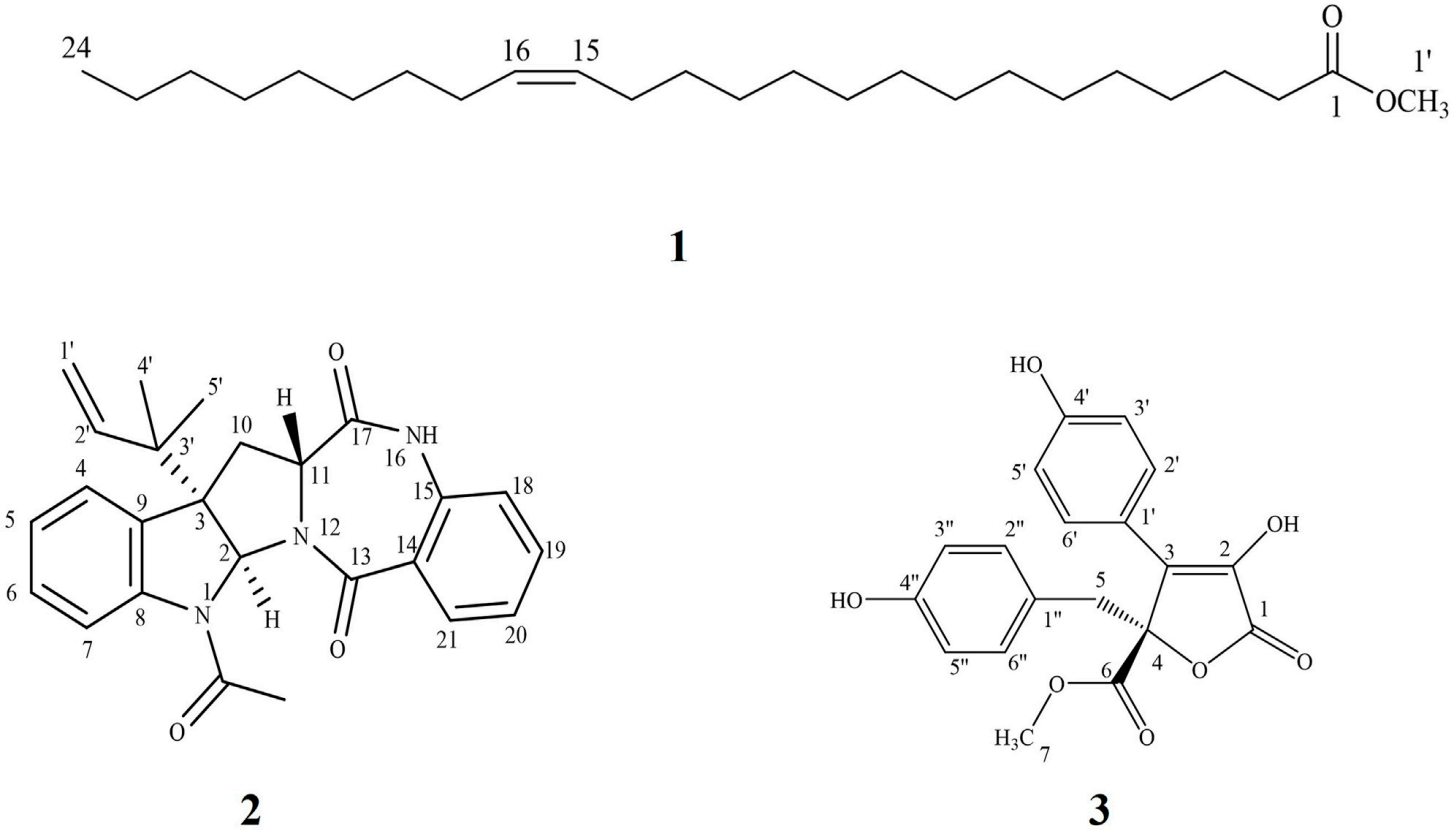
Chemical structure of isolated metabolites from *Aspergillus neoniveus.*

The ethyl acetate extract of the fungal biomass and media was preliminarily investigated by HPLC/ESI/MS/MS operating at both negative and positive modes of ionization for the metabolite analysis. Table 1 and Supplementary Figure S1 summarize the identity and chemical structures of detected molecules. Forty-five metabolites were identified based on their molecular ion peaks, fragmentation patterns, and reported literature from different *Aspergillus* species. Various chemical classes were represented by the identified metabolites including γ-butyrolactones (**15**, **20–24**, **26**, **29**, **31-37, 39,** and **41**), alkaloids (**5**, **7**, **18**, **30**, **40,** and **44**), terpenoids (**11–13**, **16-17**, and **43**), steroids (**19, 45**), dihydroisocoumarins (**8, 14**), quinone (**25**), phenolic (**1**), prenylated phenols (**2, 3, 9**), dihydrobenzofuran derivatives (**4, 10**), norneolignan (**38**), peptide **(6)**, and miscellaneous (**27**, **28** and **42**). Indeed, the pooled data strongly emphasizes the chemical diversity and active biosynthetic machinery of the isolated *A. neoniveus*. Next, we aimed at exploring the main constituents using various chromatographic techniques and evaluating the anticancer potential of the total extract and purified compounds against a panel of human prostate cancer cell lines. First, we adopted the vacuum liquid chromatography (VLC) approach using silica gel 60 as a preliminary fractionation step. Pooled fractions were grouped based on their chromatographic behavior on TLC. Each collective fraction was further purified on multiple silica gel columns using mixtures of hexane and EtOAc as mobile phase to afford three major compounds **1–3** (Figure 1, Supplementary Figure S26)**,** which are reported for the first time from the fungus *A. neoniveus.* The extensive spectral analyses of purified compounds using 1 and 2D NMR and ESI mass spectrometry revealed the chemical identity of each metabolite.

#### 3.2.1 Compound 1

It was isolated as yellow oil. Its ESI-MS showed a quasi-molecular ion peak at *m/z* 381.2989 [M + H]^+^corresponding to the molecular formula C_24_H_46_O_2_ and representing two units of unsaturation. ^1^H NMR data displayed a multiplet signal at δ_H_ 5.31 assigned for the two olefinic protons (H-15 and H-16). A triplet signal at δ_H_ 2.27 (H-2) could be assigned for the α- methylene protons of carboxylic acid derivatives. A four protons multiplet signal at δ_H_ 1.98 (H-14 and H-17) is assigned to the two methylene groups adjacent to the double bond. Moreover, a series of proton signals at δ_H_1.59 (m, H-3) and at δ_H_ 1.59-1.28 (32 H m, H-4–13 and H-18–23) supported the suggestion that the structure is a long chain fatty acid. In addition, the ^1^H NMR spectrum showed a triplet signal at δ_H_ 0.86 for a terminal methyl group and a singlet signal at δ_H_ 3.63 corresponding to a methoxy group. ^1^H–^1^H COSY spectrum showed a correlation between olefinic protons signal at δ_H_ 5.31 with proton signal at δ_H_ 1.98 (H-14 and H-17), also the correlation between H-2 (δ_H_ 2.27) with H-3 (δ_H_1.59). The ^13^C NMR, APT, and HMQC spectra of **1** revealed the presence of a carboxyl ester at δ_C_ 173.9, two olefinic carbons at δ_C_ 129.9, and 129.6 for C-15 and C-16, respectively. Moreover, the spectrum showed two carbon signals at δ_C_ 51.2 (C-1′) and 13.9 (C-24) for the methyl ester and the terminal methyl group, respectively. Furthermore, a series of characteristic secondary carbon signals as C-2 (δ_C_ 33.9), C-3 (δ_C_ 24.9), C-4(δ_C_ 31.9), C-14 (δ_C_ 27.1), C-17(δ_C_ 27.1), C-22 (δ_C_ 31.6) and C-23 (δ_C_ 22.6) were shown as a part of long chain aliphatic carbons. The HMBC data showed a correlation between different protons and carbons of the structure among which were the correlation between α- methylene protons of a carboxylic acid, H-2 (δ_H_ 2.27) with C-1 (δ_C_ 173.9), C-3 (δ_C_ 24.9) and C-4 (31.9) and that between the two olefinic protons H-15 and H-16 (δ_H_ 5.31) with C-14 (δ_C_ 27.1) and C-17 (δ_C_ 27.1), which all support the spectral assignments. Moreover, the correlation between the terminal methyl group (δ_H_ 0.86) with C-22 (δ_C_ 31.9) and C-23 (δ_C_ 22.6) and that between the OCH_3_ group (δ_H_ 3.63) with C-1 (δ_C_ 173.9) evidenced the fatty acid ester nature of compound **1**. Therefore, based on the analysis of the spectroscopic data and comparison with the previously reported data (Permana and LajisAimi, 2011; Ahmed et al., 2006) compound **1** was identified as nervonic acid methyl ester which was isolated for the first time from endophytic *Aspergillus* species.

#### 3.2.2 Compound 2

**FIGURE 2:**
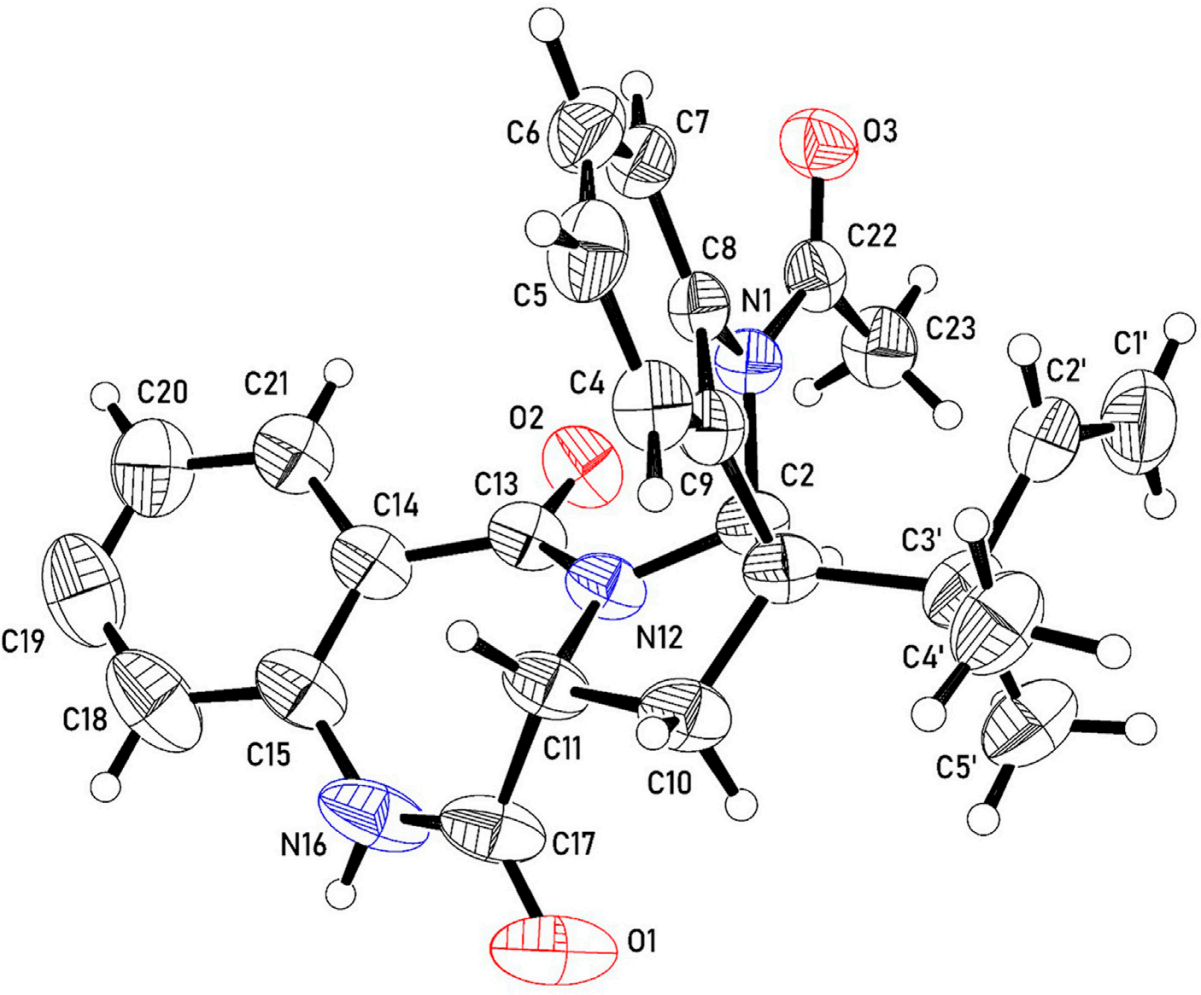
A molecule from the crystal structure of 2 showing 50% atomic displacement ellipsoids.

The ^13^C NMR, DEPT and HMQC spectra of **2** revealed the presence of 25 carbons categorized as follow: one acetyl and two amide carbonyls at δ_C_ 170.6, 169.6 and 166.6 for COCH_3_, C-17 and C-13 respectively; four quaternary *sp* ^*2*^ carbons at δ_C_ 142.0 (C-8), 133.8 (C-9), 133.8 (C-15) and 127.5 (C-14); nine methine *sp2* at δ_C_ 143.3 (C-2'), 132.3(C-19), 131.0 (C-20), 128.9 (C-6), 125.4(C-18), 124.3(C-7), 124.0 (C-5), 120.6 (C-21) and 119.2(C-4); one methylene sp2 (δ_C_ 114.2, C-1'); two quaternary sp3 at δ_C_ 60.2 (C-3) and δ_C_ 40.7(C-3'); two methine sp3 at δ_C_ 82.1 and 56.6 for C- 2 and C- 11, respectively; one methylene sp3 at δ_C_ 30.6 for C-10 and three tertiary methyl group at δ_C_ 24.1, 22.9 and 22.5 for COCH_3_, C-5' and C-4', respectively. Guided by the ^1^H NMR and HMQC spectra, eight protons in the aromatic region at δ_H_ 8.01 (m*,* H-4; δ_C_ 119.2); δ_H_ 7.67 (dd, *J* = 7.67, 0.8, H-20; δ_C_ 131.0); δ_H_ 7.39 (ddd, *J* = 8.4, 8.4, 1.2 Hz; H-19, δ_C_ 132.3); δ_H_ 7.29 (m, H-6; δ_C_ 128.9); δ_H_ 7.26 (dd, *J* = 7.6, 2.4 Hz, H-7; δ_C_ 124.3), δ_H_ 7.15 (m, H-18; δ_C_ 125.4); δ_H_ 7.08 (m, H-5; δ_C_ 124.0) and δ_H_ 6.96 (d, *J* = 8.0 Hz, H-21; δ_C_ 120.6) were assigned with their attached carbons. In addition, one doublet proton at δ_H_ 5.13 (*br*d, H-1'; δ_C_ 114.2), four doublet doublets at δ_H_ 5.89 (dd, *J* = 17.6, 10.4 Hz, H-2'; δ_C_ 143.3), δ_H_ 3.99 (dd, *J* = 8.4, 8.0, H-11; δ_C_ 56.6), δ_H_ 3.39 (dd, *J* = 13.6, 8.4, H-10; δ_C_ 30.6) and δ_H_ 2.44 (dd, *J* = 13.6, 8.4, H-10; δ_C_ 30.6) and one doublet at δ_H_ 5.10 (ddd, *J* = 14.4, 11.6, 2.8, H-1'; δ_C_ 114.2) were elucidated. Moreover, one singlet signal at δ_H_ 5.94 (*s*, H-2; δ_C_ 82.1), three methyl singlets at δ_H_ 1.19 (H-4', δ_C_ 22.5), 0.99 (H-5', δ_C_ 22.9) and δ_H_ 2.61 (COCH_3_, δ_C_ 24.1) were correlated with their carbons. The ^1^H–^1^H COSY spectrum displayed cross-peak correlations between H-18 (δ_H_ 7.15) and H-20 (δ_H_ 7.67), H-19 (δ_H_ 7.39), and H-21 (δ_H_ 6.96). These correlations together with ^1^H NMR, ^13^C NMR, and HMQC revealed the presence of the first 1, 2-disubstituted benzene ring. Moreover, it was found that the benzene ring is fused to the 1,4-bezodiazepane-3,7-dione ring resulting in the formation of 3,4-dihydro-1*H*-1,4-benzodiazepane-2,5-dione portion. This finding was confirmed by HMBC correlation which displays a correlation from H-18 (δ_H_ 7.15) to C-14 (δ_C_ 127.5), C-15 (δ_C_ 133.8), and C-21(δ_C_ 120.6), and correlation from H-19 (δ_H_ 7.39) to C-15 (δ_C_ 133.8), C-18 (δ_C_ 125.4), and C-20 (δ_C_ 131.0), from H-20 (δ_H_ 7.67) to C-15 (δ_C_ 133.8) and C-13 (δ_C_ 166.6), that from H-21 (δ_H_ 6.96) to C-18 (δ_C_ 125.4) and C-13(δ_C_ 166.6). Furthermore, the presence of a second 1,2-disubstituted benzene ring was confirmed from ^1^HNMR, ^13^CNMR, and HMQC in addition to the COSY spectrum which exhibited the correlations from H-4 (δ_H_ 8.1) to H-6 (δ_H_ 7.29). Moreover, this second 1,2-disubstituted benzene ring was attached to 2,3-dihydro-1-acetyl-indole moiety from HMBC correlations which revealed correlations from H-4 (δ_H_ 8.1) to C-9 (δ_C_ 133.8) and C-3' (δ_C_ 40.7) from H-5 (δ_H_ 7.08) to C- 4 (δ_C_ 119.2). C- 6 (δ_C_ 128.9) and C- 9 (δ_C_ 133.8), from H-6 (δ_H_ 7.29) to C- 7 (δ_C_ 124.3) and C-8 (δ_C_ 142.0), from H-7 (δ_H_ 7.26) to C-6 (δ_C_ 128.9) and C-8 (δ_C_ 142.0), from H-2 (δ_H_ 5.93) to C-8 (δ_C_ 142.0), C-9 (δ_C_ 133.8), C-3' (δ_C_ 40.7) and C-4' (δ_C_ 22.4). The presence of *the N*-acetyl group was confirmed by the correlation of CH_3-_Ac (δ_H_ 2.61) to δ_C_ 170.6 of the carboxamide moiety. Therefore, from the analysis of the above data, the coupling system of the aromatic protons observed in the H-H COSY and HMBC spectra (Table 3) revealed the presence of a couple of 1,2-disubstituted benzene rings. Moreover, the HMBC spectrum indicated that one of the 1,2-disubstituted benzene rings is a part of a 2,3-dihydro-1-acetyl-indole moiety, while the second one is a part of a 3,4-dihydro-1*H*-1,4-benzodiazepine-2,5-dione portion. The 2,3-dihydro-*H*-indole and the 3,4-dihydro-1*H*-1,4-benzodiazepine-2,5-dione portions were linked together through a pyrrolidine ring, which was confirmed from the HMBC correlation from H-10a (δ_H_ 2.44) to C-3 (δ_C_ 60.2), C-2 (δ_C_ 82.1) and C-9 (δ_C_ 133.8) and that between 10b (δ_H_ 3.39) and C-3' (δ_C_ 40.7), C-3 (δ_C_ 60.2), C-9 (δ_C_ 133.8), C-11 (δ_C_ 56.6) and C-17(δ_C_ 169.6), in addition to a correlation between H-11 (δ_H_ 3.91) and C-10 (δ_C_ 30.6). Moreover, the presence of 2-methylbut-3-en-2-yl group in the structure of compound **2** was established from ^1^HNMR, ^13^CNMR, and HMQC spectra. Its position was confirmed at C-3 from HMBC spectrum which display correlation from H-2 (δH 5.94 to the C-3' (δ_C_ 40.7), C-4' (δ_C_ 22.5), and that from CH_3_- 5′ (δ_H_ 0.99) to C-3 (δ_C_ 60.2), C-2′ (δ_C_ 60.2), C-3' (δ_C_ 40.7) and C-4' (δ_C_ 22.5), from CH_3_- 4′ (δH.1.19) to C-3 (δ_C_ 60.2), C-2′ (δ_C_ 60.2), C-3' (δ_C_ 40.7) and C-5' (δ_C_ 22.9), from H-2′ (δ_H_ 5.89) to C-3' (δ_C_ 40.7) and C-5' (δ_C_ 22.9) and that between H-1’a (δ_H_ 5.10) and H-1’b (δ_H_ 5.13) to C-2′ C-3' (δ_C_ 40.7). Additional structural confirmation of compound **2** was also established from the negative ESI/MS which displayed a molecular ion peak at 414.3071[M-H]ˉ. Furthermore, the three-dimensional structure and the absolute configuration of asymmetric centers at C-2, C-3, and C-11 were deduced from the single-crystal X-ray structure. As depicted in Figure 2, each of the two fused five-membered pyrrolidine rings (C-2, C-3, C-9, C-8, N-1, and C-2, C-3, C-10, C-11, N-12) is in enveloping conformation and the seven-membered diazepane ring is in boat conformation. The butene group (C-1', C-2') is oriented roughly parallel to the acyl group (C-22, C-23, O-3) with a torsion angle C-1',C-2',C-3',C-3 of -113.7(3)°. Other crystal structures containing **2** have also been reported and they show differences in molecular conformation. In one structure (Lin et al., 2020), the ring conformations and the orientations of the *N*-H hydrogen bond donor and carbonyl acceptors are similar to those observed in this work. However, the orientation of the butene group is almost perpendicular to the acyl group, with a torsion angle of C-1', C-2', C-3', and C-3 of 121.5°. In the second reported crystal structure (Rank, et al., 2006), the diazepane ring is inverted and thus the relative orientation of the *N*-H hydrogen bond donor and carbonyl acceptors is modified relative to the structure in this work. The location of the butene group is also different through rotation of the methyl butene group about the C-3, C-3’ bond. Thus, and in accordance with the X-ray diffraction data, compound **2** was assigned as (2*S*, 3*S*, 11*R*) acetylaszonalenin.

#### 3.2.3 Compound 3

It was isolated as buff amorphous powder. Its NMR spectra displayed two *ortho* doublets in the aromatic region at δ_H_ 7.62 and 6.90 (*J* = 8.4 Hz) for H-2'/6' (δ_C_ 129.1) and H-3'/5' (δ_C_ 115.4), respectively which confirms the presence of 1',4'-disubstituted aromatic ring. Moreover, two aromatic signals at δ_H_ 6.67 (**2´´/6´´**, δ_C_ 131.6), 6.55 (3´´/5´´, δ_C_ 114.3) which support the presence of another 1**´´**,4**´´**-disubstituted aromatic ring. Furthermore, the ^1^H NMR spectrum showed multiplet signal at ≈ δ_H_ 3.49 and 3.37 (δ_C_ 38.4) which supports the presence of methylene group attached to the sp2 system (CH_2_-5, δ_C_ 38.1`). Moreover, ^13^C NMR and APT spectra confirmed the presence of a lactone ring, deduced through the presence of carbonyl carbon at δ_C_ 169.3 (C-1) together with C-3 (δ_C_ 127.9) and C-4 (δ_C_ 85.6). A carboxylic acid methyl ester group was suggested by the presence of carbonyl carbon at δ_C_ 170.2 (C-6) with the methoxy group at δ_C_ 52.7 (δ_H_ 3.78, *O*CH_3_). These findings were confirmed by an extensive analysis of correlations of the HMBC spectrum. The first 1,4-disubstituted aromatic ring was confirmed by the correlation from H-2'/6' (δ_H_ 7.62) with C-3 (δ_C_127.9) and C-4' (δ_C_ 157.8) and C-3'/5' (δ_C_ 115.4) and that from H-3'/5' (δ_H_ 6.90) with C-1' (δ_C_ 121.8) and C-4' (δ_C_ 157.8). Moreover, the second 1,4-disubstituted aromatic ring was established by the correlation from H- 2´´/6´´ (δ_H_ 6.67) to C**-**3ˊˊ/5ˊˊ (δ_C_ 114.3), C -4ˊˊ (δ_C_ 155.9) and C-5 (δ_C_ 38.1) and that from H- 3´´/5´´ (δ_H_ 6.55) to C-1ˊˊ (δ_C_ 123.9), 2ˊˊ/6ˊˊ (δ_C_ 131.3), 4ˊˊ (δ_C_ 155.9). Furthermore, the attachment between the lactone ring and the first aromatic ring was confirmed from the correlation between the H-2'/6' (δ_H_ 7.62) with C-3 (δ_C_127.9) and its attachment with the second aromatic ring through the CH_2_-5 bridge was established through the correlation from H-5 (δ_H_ 3.49 and 3.37) to C-3 (δ_C_ 127.9), C-4 (δ_C_ 85.6), 6 (δ_C_ 170.2), 1ˊˊ(δ_C_ 123.9), 2ˊˊ/6´´(δ_C_ 131.3). Moreover, the position of the *O*CH_3_-7 was confirmed by its correlation with C-6 (δ_C_ 170.2). From the spectral analysis of compound **3**, along with its quasi-molecular ion peak at *m/z* 355.2861[M-H]^-^ correlated with a molecular formula C_24_H_24_O_7_, and in the light of previously reported data (Yang et al., 2018), the chemical identity of **3** was established as butyrolactone II, which is isolated for the first time from the fungus *A. neoniveus*.

### 3.3 Anticancer activity

#### 3.3.1 Antiproliferative effect of microbial extract and purified metabolites against human prostate cancer cells

**FIGURE 3:**
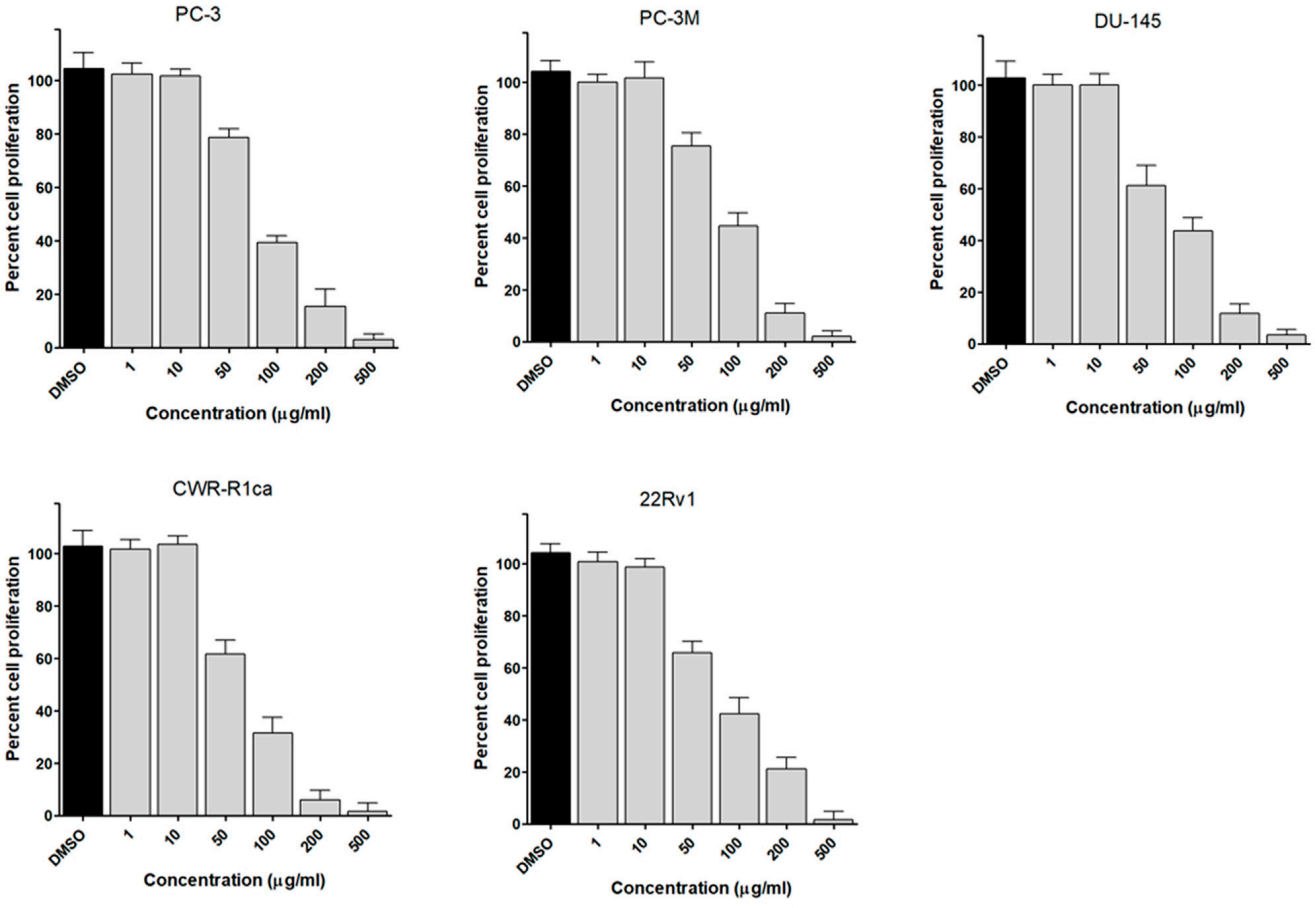
Effect of the microbial extract on the proliferation of different human prostate cancer cell lines (PC-3, PC-3M, DU-145, CWR-R1ca, and 22Rv1). Data points represent mean percent cell proliferation (± SD) at indicated concentrations.

**FIGURE 4:**
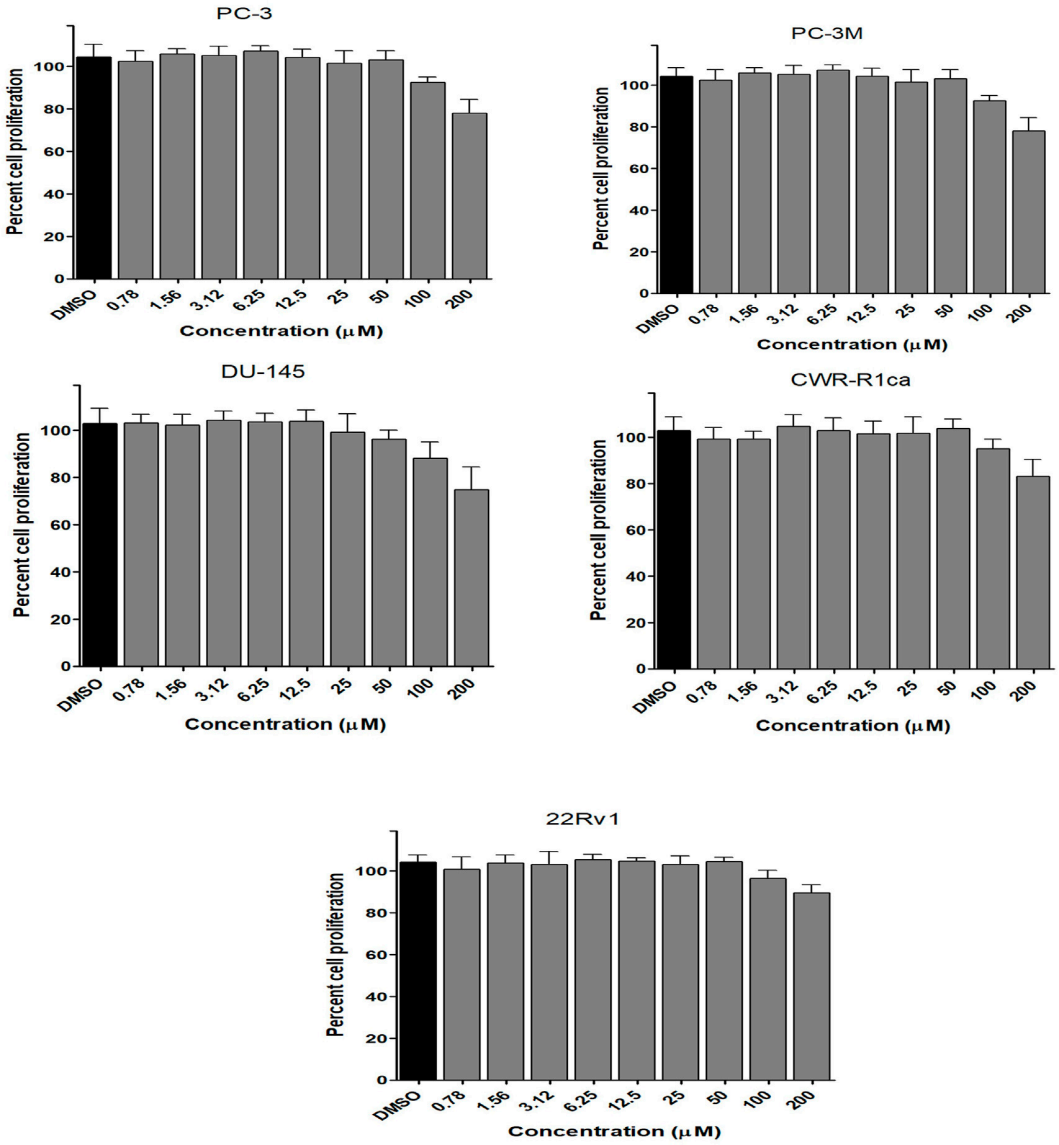
Effect of the compound 1 on the proliferation of different human prostate cancer cell lines (PC-3, PC-3M, DU-145, CWR-R1ca, and 22Rv1). Data points represent mean percent cell proliferation (± SD) at indicated concentrations.

**FIGURE 5:**
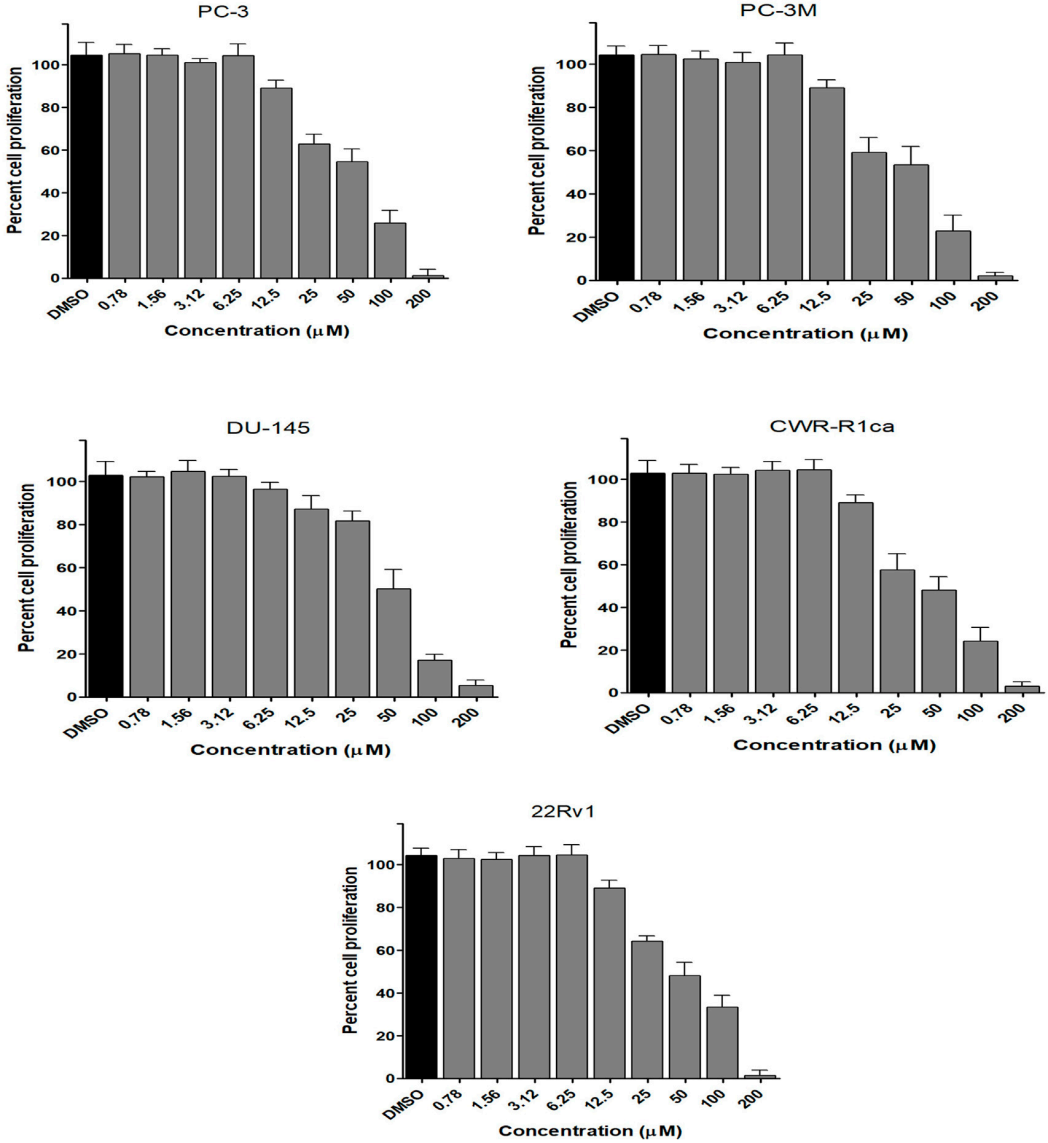
Effect of the compound 2 on the proliferation of different human prostate cancer cell lines (PC-3, PC-3M, DU-145, CWR-R1ca, and 22Rv1). Data points represent mean percent cell proliferation (± SD) at indicated concentrations.

**FIGURE 6:**
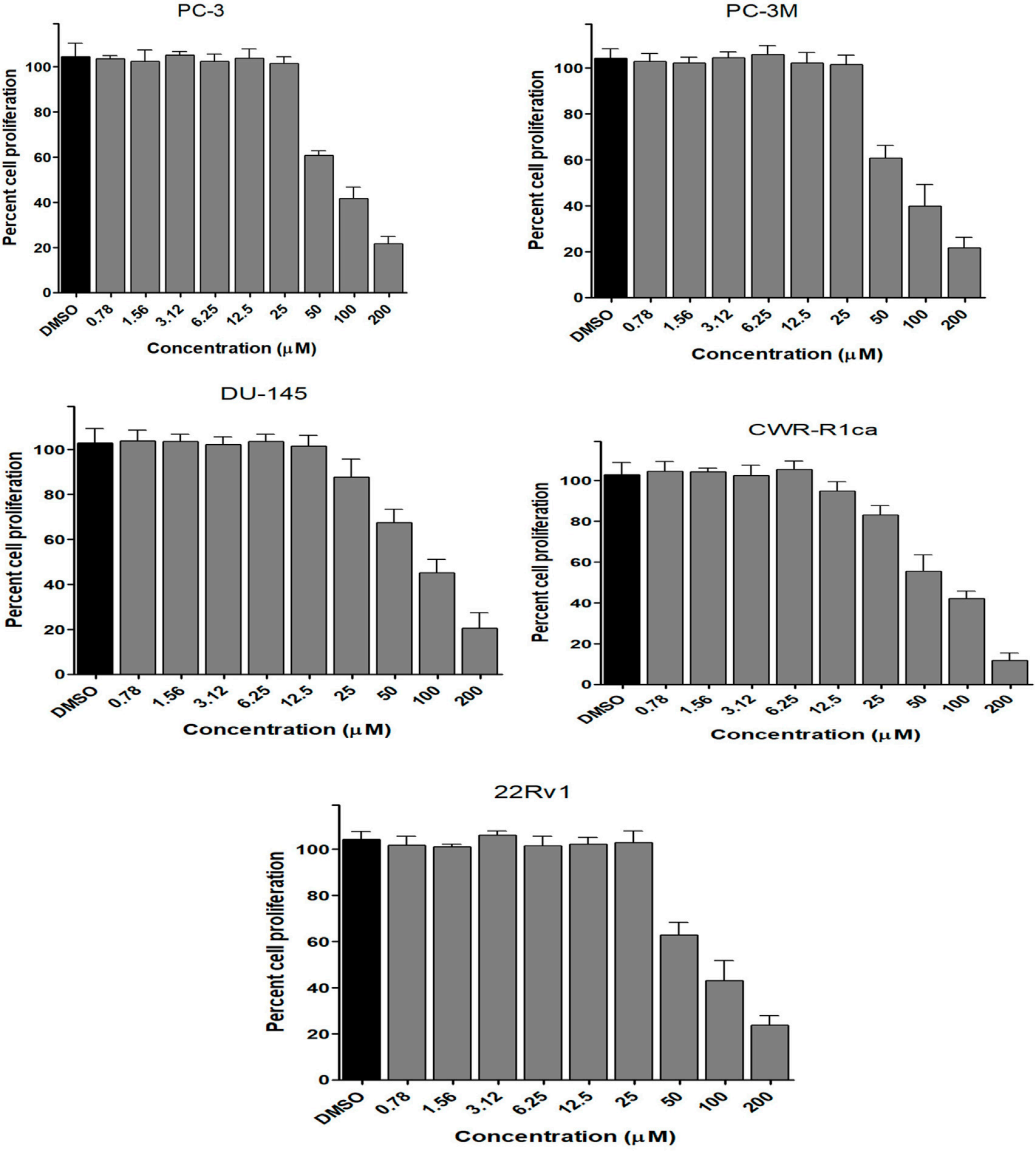
Effect of the compound 3 on the proliferation of different human prostate cancer cell lines (PC-3, PC-3M, DU-145, CWR-R1ca, and 22Rv1). Data points represent mean percent cell proliferation (± SD) at indicated concentrations.

To assess the effect of the ethyl acetate extract of *A. neonevius* rice media and grown mycelia on the proliferation of a panel of human prostate cancer cells with different phenotypes and molecular signatures, 10 mg/ml DMSO stock solution of the microbial extract was used to prepare a set of final treatment concentrations ranging approximately from 500 to 1 μg/ml in RPMI-1640 culture medium. Percent cell proliferation at each concentration was calculated relative to the DMSO-treated control cells (100% cell proliferation). As depicted in Figure 3, the microbial extract showed a significant growth inhibition against all tested prostate cancer cells, albeit with different degrees of potencies. For instance, the most sensitive cell line to the microbial extract was the castration-resistant CWR-R1ca with a calculated IC_50_ value of 79.2 μg/ml (Table 6). In contrast, the least sensitive prostate cancer cells were the metastatic PC-3M, followed by PC-3, DU-145, and 22Rv1, in that order. The calculated IC_50_ values for all cell lines are listed in Table 6. The promising antiproliferative activity of the crude microbial extract encouraged us to investigate the metabolic profile of the microbial extract aiming to isolate the main metabolites and test their ability to slow down the proliferation capacity of various prostate cancer cells. As shown in Figures 5, 6, only **2** and **3** significantly inhibited the proliferation of all prostate cancer cells in a dose-dependent manner. Meanwhile, **1** (Figure 4) showed just a minimal inhibitory effect up to 200 µM. Based on the effect of cell growth pattern and calculated IC_50_ values, **2** showed a superior antiproliferative effect than **3** and was maximally potent against the castration-resistant CWR-R1ca cells with a calculated IC_50_ value of 61.4 µM. Notably, castration-resistant prostate cancer is the most aggressive phenotype with very limited therapeutic options. Hence, our preliminary data support the future testing of **2** in animal models to explore its efficacy on the tumorigenic growth of castration-resistant prostate cancer cells in relevant animal models.

**TABLE 6:** IC_50_ (± SD) values of ethyl acetate extract and compounds 1-3 in the cell proliferation assay against different human prostate cancer cell lines.

Cell line	PC-3	PC-3M	DU-145	CWR-R1ca	22Rv1
Extract (µg/ml)	122.4 ± 9.6	135.6 ± 8.1	96.6 ± 7.2	79.2 ± 4.5	108.7 ± 6.3
Metabolites (µM)					
**1**	>200	>200	>200	>200	>200
**2**	87.7 ± 6.2	76.4 ± 4.1	84.2 ± 7.4	61.4 ± 5.5	74.8 ± 6.3
**3**	161.7 ± 7.6	152.8 ± 8.5	166.5 ± 10.7	114.8 ± 11.5	173.4 ± 8.9

#### 3.3.2 Antimigratory effect of microbial extract and purified metabolites (1–3) against different human prostate cancer cells

**FIGURE 7:**
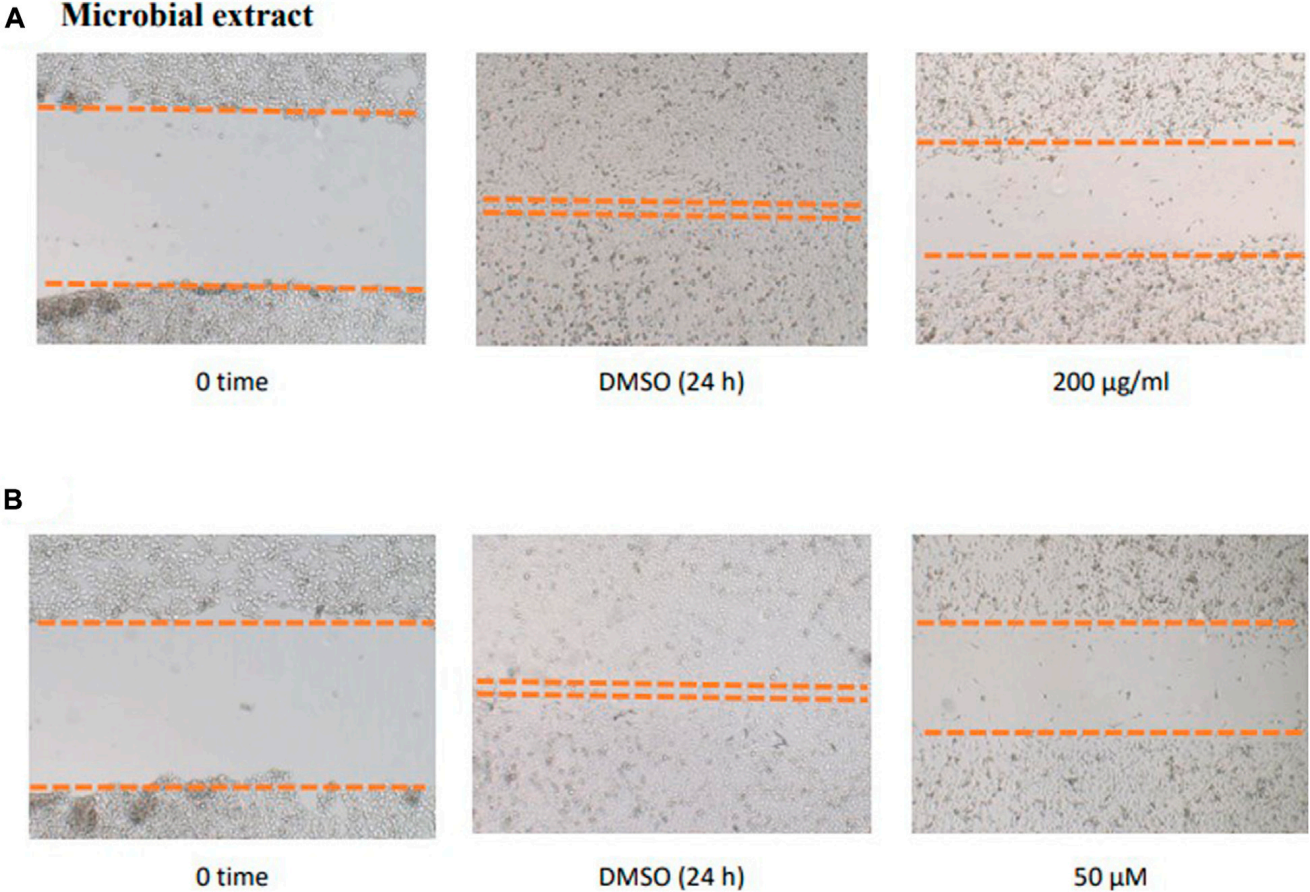
Effect of the microbial extract **(A)** and acetylaszonalenin **(B)** of the motility of human PC-3 prostate cancer cells in the wound healing assay. Microphotographs represent the wound at the creation time (t0) and 24 h post treatment in DMSO- (negative control) and acetylaszonalenin-treated wells.

To study the potential effect of the microbial extract on the *in vitro* 2D migration of different prostate cancer cell lines, scratched cell monolayers were incubated with different concentrations of the extract (200–1 μg/ml) for 24–48 h and monitored for wound closure in vehicle control-treated cells. Data revealed the significant inhibition of cell motility compared to control cells at the end of the experiment (Figure 7) with a calculated IC_50_ value of 41.7 μg/ml for the most sensitive metastatic PC-3 cells. The IC_50_ values for other cells are shown in Table 7. These promising data justified testing of pure isolated metabolites under the same *in vitro* platform. Results revealed the significant inhibitory effect of **2** and **3** on the motility of the different prostate cancer cells, showing that **2** is more potent. Furthermore, **2** was selectively potent against the highly metastatic PC-3 prostate cancer cells with a calculated IC_50_ value of 18.5 µM. However, **1** did not show a promising inhibitory effect on the *in vitro* wound closure at comparable concentrations with **2** and **3**. These promising results illustrated the potential inhibitory effect of the isolated compounds and support the future investigation of their antimetastatic potentiality in relevant prostate cancer animal models.

**TABLE 7:** IC_50_ (± SD) values of ethyl acetate extract and compounds 1-3 against different prostate cancer cell lines in the cell migration assay.

Cell line	PC-3	PC-3M	DU-145	CWR-R1ca	22Rv1
Extract (µg/ml)	41.7	86.6	98.1	102.4	78.5
Metabolites (µM)					
**1**	>100	>100	>100	>100	>100
**2**	18.5 ± 2.1	27.3 ± 3.1	43.2 ± 2.2	41.8 ± 3.7	33.6 ± 3.1
**3**	67.2 ± 4.5	>100	>100	58.2 ± 4.5	49 ± 3.7

#### 3.3.3 Anti-clonogenic effect of compound 2 against human prostate cancer cells

**FIGURE 8:**
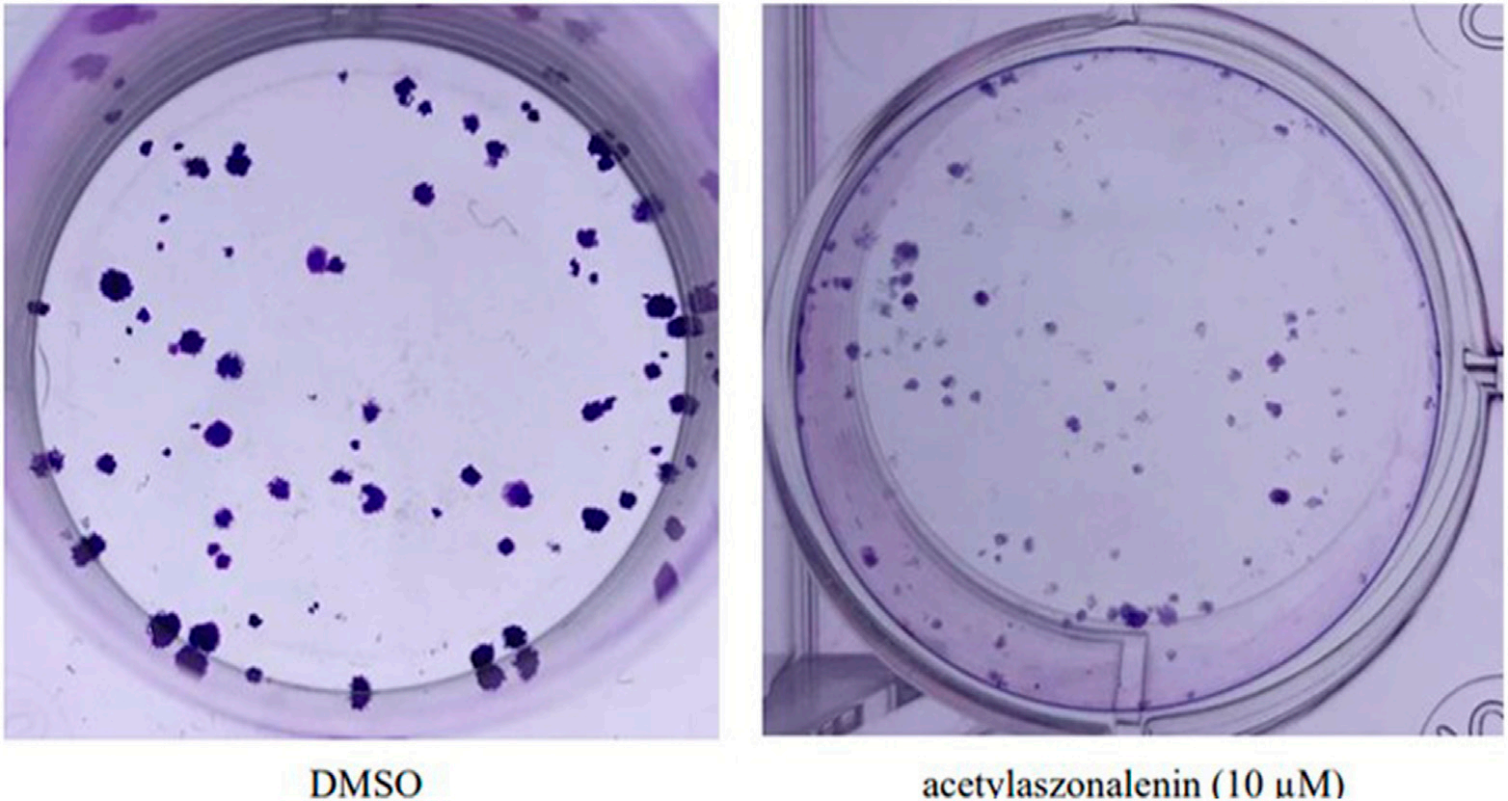
Effect of acetylaszonalenin (2) on the clonognic survival of 22Rv1 human prostate cancer cells. Photographs show numerous cell colonies in DMSO-treated cells (negative control), while minimal colonies are seen at 10 µM of acetylaszonalenin indicating a significant suppression of clonogenic growth.

The pooled data in cell proliferation and motility assays (see above) proved the superiority of **2** in different anticancer screening platforms. Hence, **2** was selected for efficacy evaluation against the clonogenic growth and survival of different prostate cancer cells. In principle, the ability of a single cell to grow and form a cluster of more than 50 cells is known as clonogenic growth. It closely mimics the *in vivo* scenario where single cells are transformed into tumorigenic cells and start colonizing and forming tumorigenic foci. Thus, prostate cancer cells were treated with different concentrations of **2** over 15–20 days, with periodical changing of the treatment media every 72 h. The evaluation of the results revealed the significant clonogenicity inhibition of **2** against different cancer cells with the low micromolar level of activity against the most sensitive 22RV1 cells (Figure 8). Compound **2** showed IC_50_ values ranging from 7 to 22 µM against the tested cancer cell lines, which are listed in Table 8. These promising data augment the previous anticancer assays and promote **2** for further biological evaluation as a promising natural products-based anticancer hit.

**TABLE 8:** IC_50_ (± SD) values compound 2 in the cell clonogenic survival assay against different prostate cancer cell lines.

Cell line	PC-3	PC-3M	DU-145	CWR-R1ca	22Rv1
**Compound 2**	16.4 ± 2.4	22.5 ± 5.1	18.6 ± 3.3	13.1 ± 1.9	7.8 µM ± 1.1

### 3.4 Computational prediction of molecular target(s)

**FIGURE 9:**
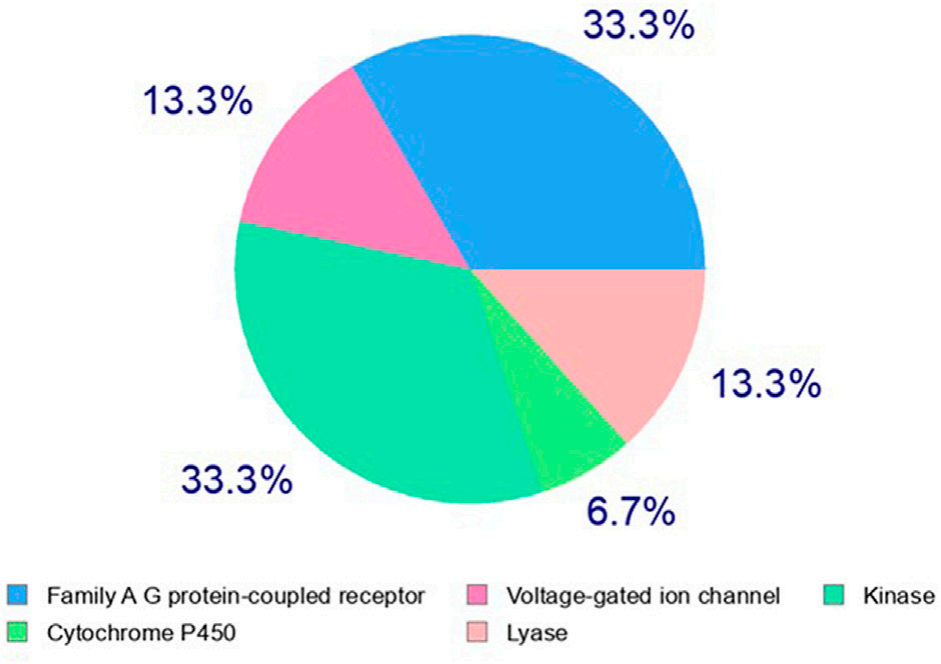
Computational prediction of potential molecular targets for acetylaszonalenin (2) by Swiss Target Prediction. Pie chart represents the prediction percentages for each class of human biological target proteins.

Identification of the molecular mechanism of action is a key milestone in early anticancer drug discovery. The anticancer screening assays detecting changes in tumor cell phenotypes such as cell proliferation, migration, and invasion, upon treatment with test molecules, are of great utility for hit identification and validation. However, they are of limited capability to identify the potential macromolecular targets underlying the pharmacological response. The experimental target identification can be accomplished using various approaches (such as proteomic analyses and affinity chromatography) which are frequently time and cost expensive. On the other hand, computational analyses can rapidly predict primary targets for bioactive hits even at no cost in many cases. Three main strategies are implemented to accomplish target hitting, including ligand-based, structure-based, and hybrid approaches (Sydow et al., 2019). In the ligand-based approaches, structure similarity is principally implemented in the search methodology, which relies on the notion that similar structures possess a similar biological activity. Herein we utilized the web-based Swiss Target identification interface to predict a potential macromolecular target for acetylaszonalenin (**2)**. MDL Mol file of **2** was generated and submitted for computational search with *Homo sapiens* set as relevant species. The general class of the macromolecular targets (retrieved from ChEMBL data base for drug-like molecules) identified by the ligand similarity search approach were scored based on the two-dimensional and three-dimensional similarity values to the nearest experimentally active molecule. Furthermore, the specific targets of each class or family hits were ranked according to the highest given probability values, in additional to the number of available bioactive 2D and 3D structures for each specific macromolecular target. Our results from the online computational search demonstrated that G-protein coupled receptors family (GPCRs) and kinases could be potential target classes for acetylaszonalenin with prediction percentages of 33.3 for both class targets (Figure 9). Meanwhile, lyases and voltage-gated ion channels are less likely with a prediction of 13.3%, and cytochrome P450s are most less likely (6.7%) to be affected by acetylaszonalenin. Furthermore, a hundred protein targets were identified, and the cannabinoid receptor type 1 (CB1) was listed at the top of the G-protein coupled receptors family of molecular targets with a probability value of 0.127, and 1070/18 identified 3D/2D bioactive known molecules after excluding the structures with values less than 0.85 in the electroshape 5D vectors (ES5D) approach for 3D similarity and 0.65 for path-based fingerprint vectors (FP2D) for 2D similarity. These seminal results inspired us to validate the computational target identification and to investigate the docking mode of acetylaszonalenin at the CB1 receptor binding cavity.

### 3.5 Molecular docking study

**FIGURE 10:**
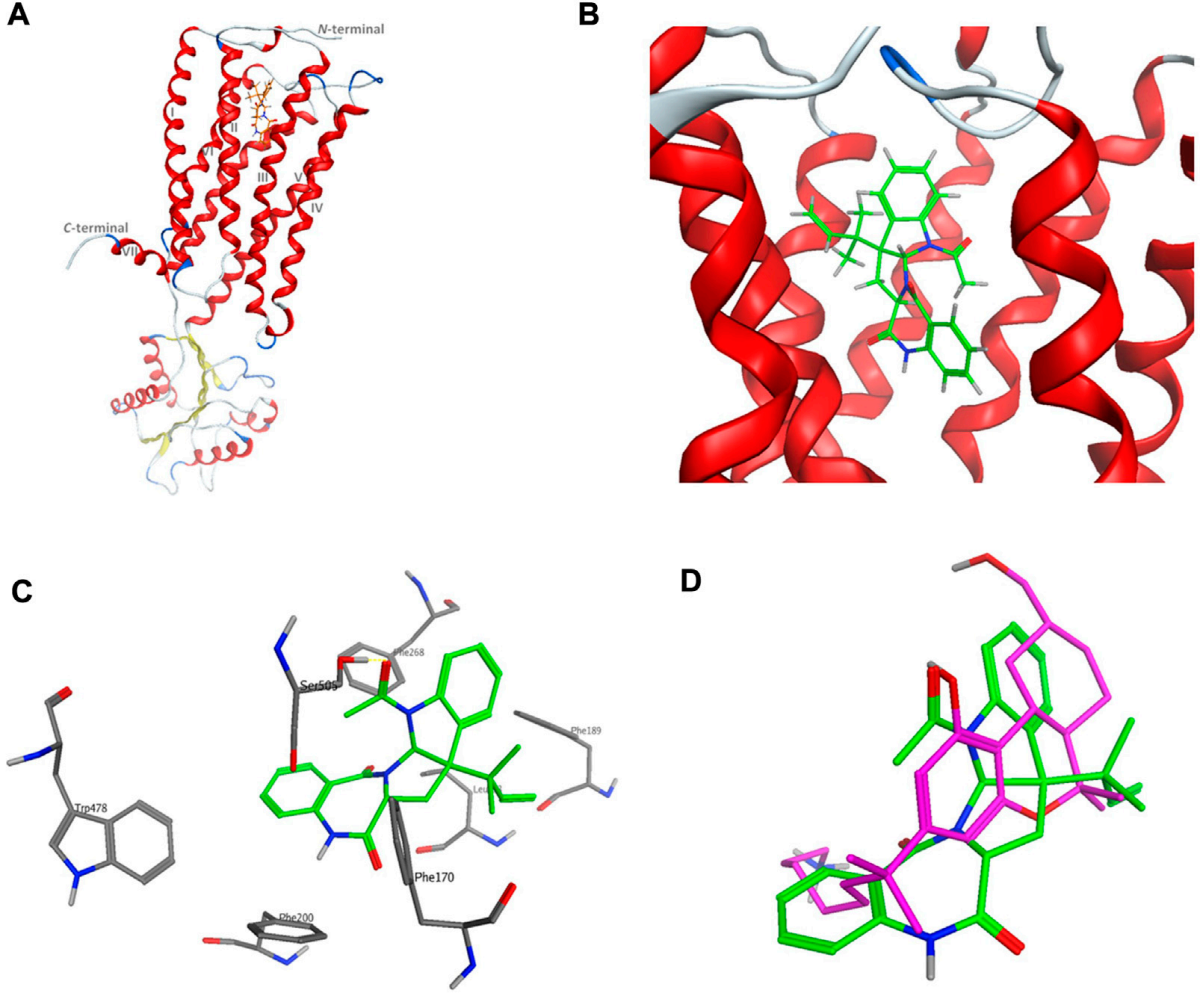
Virtual binding mode of acetylaszonalenin at the cannabinoid receptor 1 (CB1) orthosteric binding pocket. **(A)** Cartoon representation of the overall of CB1 and its ligand binding site at the extracellular face of the transmembrane helices. **(B)** Close-up of acetylaszonalenin binding pose showing its twisted conformation. **(C)** Molecular interactions of acetylaszonalenin and nearby amino acids at the orthosteric binding site. **(D)** Overlay of the cannabinoid X-ray crystallized ligand (purple) and acetylaszonalenin (green) binding poses at CB1 receptor binding pocket.

The computational target identification by the Swiss Target Prediction interface indicated that the human cannabinoid G-protein coupled receptor type 1 (CB1) is a top potential target for acetylaszonalenin (**2**). Thereafter, molecular docking studies were launched to investigate the virtual binding mode of **2** at the CB1 ligand-pocket and consequently trace significant molecular interactions. Firstly, the CB1 receptor adopted the classical seven-transmembrane fold (TM I-VII) of the G-protein coupled receptors (GPCRs) (Figure 10), with an agonist-bound conformation due to the absence of ionic lock between Arg214 and Asp338 (Shao, et al., 2016). Furthermore, the *N*-terminal domain just preceding the TM I helix was slightly distorted away from the orthosteric pocket confirming the active conformation of the GPCR. Besides, the ligand binding cavity was located at the gap between TM III and TM VII in the extracellular leaflet and lined with highly hydrophobic amino acids (such as Phe, Trp, Met, Leu, and Ile). Furthermore, the TM III/VII gap is hydrophobically evidenced by the presence of Ile119, Phe381, and Met384 residues, thus facilitating the entry of cannabinoid modulators to the deep pocket. Acetylaszonalenin (**2**) was docked at the orthosteric pocket, and low-energy poses were examined to gain insight into the potential molecular interactions. The docking results revealed that acetylaszonalenin adopted a partially extended conformation with a twisting turn at the junction between the 1,3-diazepane-2,5-dione and 2,3-dihydro-1*H*-indole, and the *N*-acetyl and 1′,1′-*gem*-dimethylpropene attached to the indoline moiety were oriented towards opposite directions (Figure 10). At the orthosteric binding cleft, acetylaszonalenin experienced multiple interactions including a hydrogen bond between its acetyl carbonyl oxygen and Ser505 at the TM helix VII, and N-H/π interaction between its 1,3-diazepane-2,5-dione NH and phenyl side-chain of Phe200 at TM helix III. Moreover, the pentacyclic core exhibited multiple hydrophobic interactions with nearby hydrophobic amino acid residues including Phe170, Phe189, Phe268, and Trp478 lining the lipophilic binding pocket (Figure 10). Furthermore, these multiple molecular contacts were also manifested in the predicted binding energy of acetylaszonalenin/CB1 interaction (-9.86 kcal/mol), which further supported the highly posing quality of the docking results. In the same sense, the predicted binding posed of acetylaszonalenin was overlaid with the experimental binding conformation of the X-ray co-crystalized ligand (AM841, a synthetic cannabinoid). Interestingly, both ligands occupied the same vicinity, where the tricyclic terpenoid ring system was laminated over the pyrrolo[2,1-c][1,3]benzodiazepine of acetylaszonalenin. Meanwhile, the indoline moiety of acetylaszonalenin partially occupied the binding groove in which the *gem*-1′,1′-dimethylheptyl moiety of the X-ray co-crystalized ligand was located. In summary, acetylaszonalenin has a high potential to modulate the CB1 receptor that is, at least in part, correlated to its anti-prostatic cancer effects.

## 4 Conclusion

Natural products have proven their significant contribution to the field of anticancer drug discovery. Microbes, including fungi, are well-documented biosynthetic producers of a plethora of chemically and biologically diverse secondary metabolites. HPLC/ESI-MS/MS analysis of the metabolite profile of *A. neoniveus* led to the identification of numerous compounds including terpenoids, alkaloids, γ-butyrolactone, and quinoids. Furthermore, nervonic methyl ester, acetylaszonalenin, and butyrolactone II were isolated from *A. neoniveus* for the first time. The absolute configuration and the single-crystal X-ray structure of the acetylaszonalenin is reported. Moreover, the microbial extract and its isolated metabolites have shown significant antiproliferative, anti-migratory, and anti-clonogenic activities against different human prostate cancer cells at low micromolar concentrations, including cells with a castration-resistance phenotype. Furthermore, the computational target prediction linked the cannabinoid G-protein coupled receptors type 1 (CB1) as a potential target underlying, at least in part, the anticancer effects of acetylaszonalenin (**2**). In addition, molecular docking experiments revealed low-energy binding poses at the CB1 receptor orthosteric pocket aided by multiple polar and hydrophobic interactions with critical amino acids. Taken together, *A. neoniveus-*derived acetylaszonalenin is a promising anticancer alkaloid, amenable for further hit-to-lead optimization aiming at the control of prostate malignancies *via* targeting CB1 receptors.

## Data Availability

The datasets presented in this study can be found in online repositories. The names of the repository/repositories and accession number(s) can be found in the article/Supplementary Material.
