# Supplementary material for: Anti-prostate cancer metabolites from the soil-derived Aspergillus neoniveus
Source: Front Pharmacol. 2022 Oct 14;13:1006062. doi: 10.3389/fphar.2022.1006062 (PMC9614250; doi:10.3389/fphar.2022.1006062)
Supplement: Supplementary file 1 [file DataSheet1.PDF]

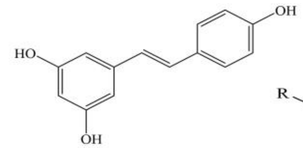

1

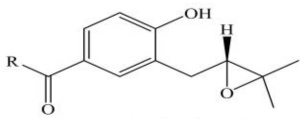

2: R = H; 9: R = OH

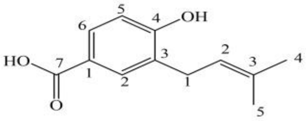

3

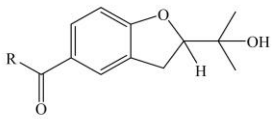

4

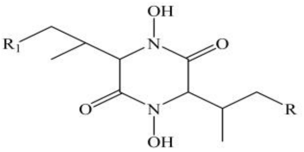

5: R = R_1=; 7: R = H, R_1= CH_3

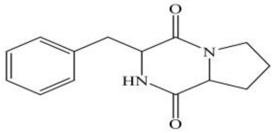

6

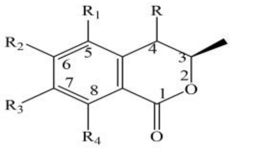

8: R = R_1 = R_3 = H, R_2 = R_4 = OCH_3
14: R = R_1 = CH_3, R_2 = R_4 = OH, R_3 = COOH

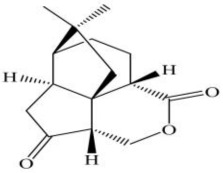

11

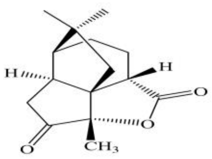

12

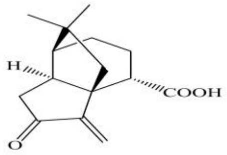

13

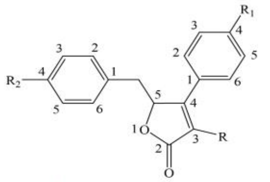

15: R = OH, R_1 = R_2 = H
14: R = OCH_3, R_1 =R_2= H

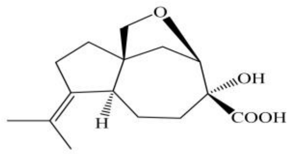

16

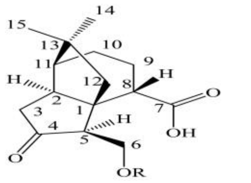

17: R = H; 43: R = CH_3

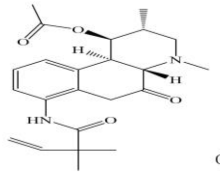

18

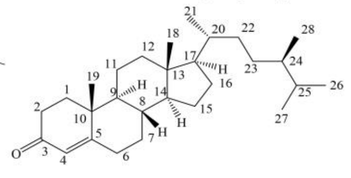

19

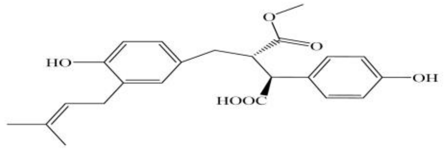

20

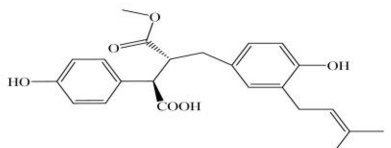

21

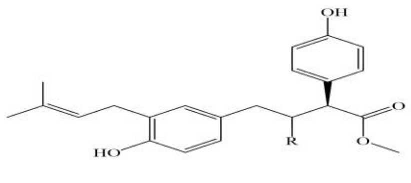

22: R = *α*-COOH; 23: R = *β*-COOH

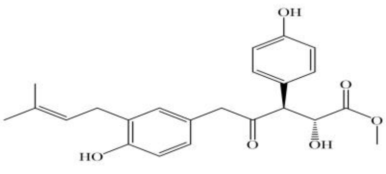

24

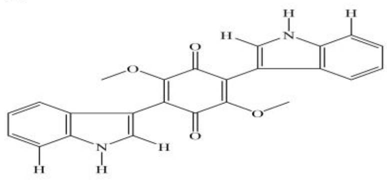

25

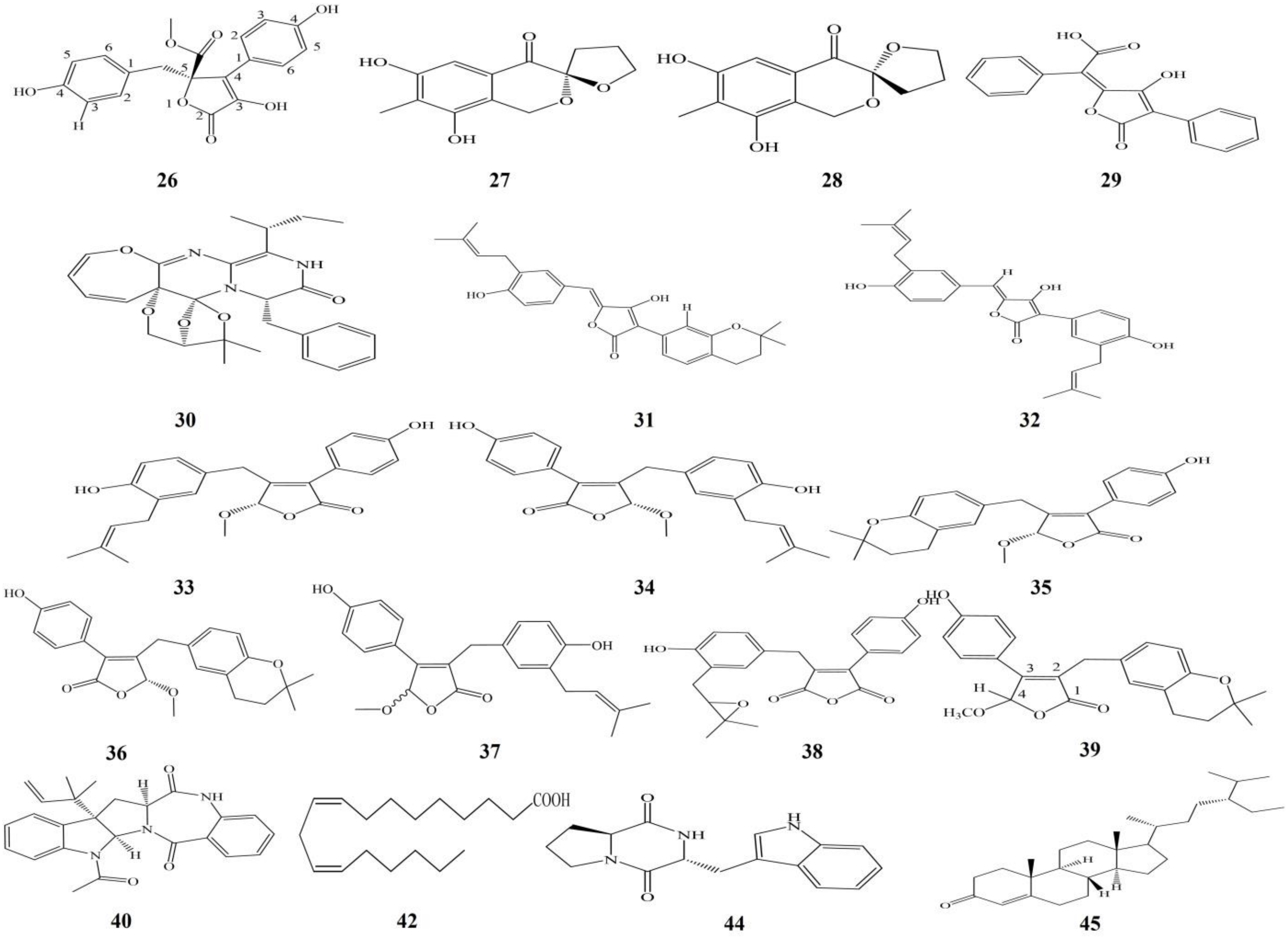

Figure 1S. Tentatively identified compounds in the ethyl acetate extract of *Aspergillus neoniveus* by HPLC/ESIMS/MS analysis.

Center for DRUG DISCOVERY RESEARCH and DEVELOPMENT
Openlynx Report -

Page 1

Sample: 518
File:D21 66
Description:MF1q

Vial:1:A,8
Date:05-Sep-2021

ID:
Time:17:18:39

Printed: Wed Sep 08 16:32:02 2021

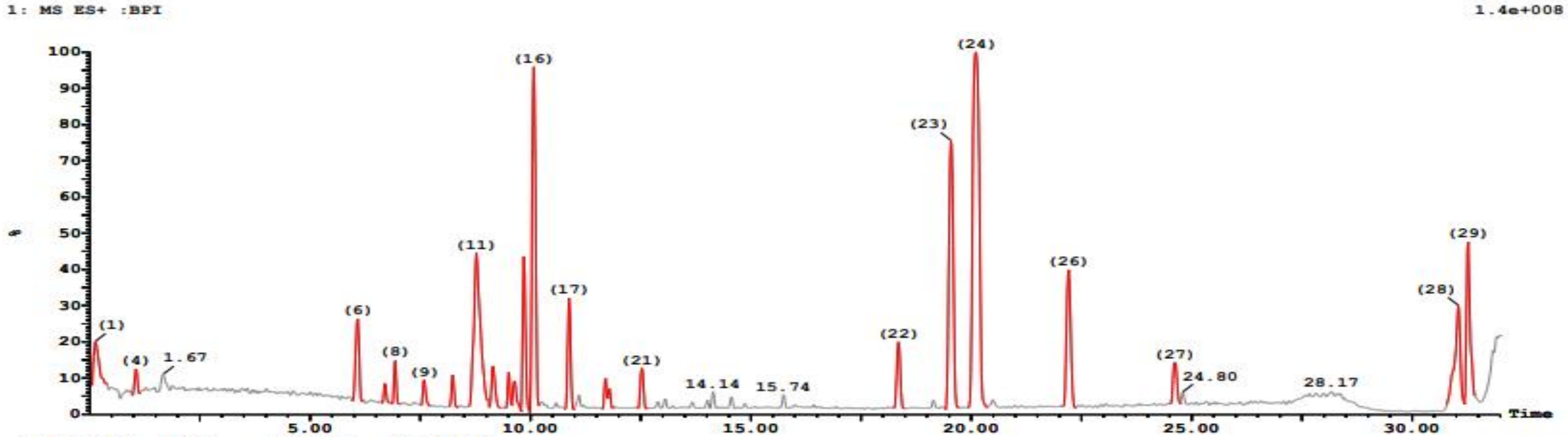

Peak Number	Time	AreaAbs	Area %Total
1	0.13	2325296.7500	2.24
4	1.05	739409.5625	0.71
6	6.08	3066719.0000	2.95
7	6.69	513783.0313	0.49
8	6.93	1052482.2500	1.01
9	7.59	748441.3750	0.72
10	8.24	841777.3750	0.81
11	8.77	9997202.0000	9.62
12	9.16	1534655.5000	1.48
13	9.50	773627.5625	0.74
14	9.64	973086.7500	0.94
15	9.85	4483150.0000	4.31
16	10.08	12608754.0000	12.13
17	10.88	3334924.0000	3.21
19	1.71	749678.8125	0.72
20	11.80	522243.2813	0.50
21	12.52	1343128.7500	1.29
22	18.36	2407707.7500	2.32
23	19.54	12080610.0000	11.62
24	20.11	24398272.0000	23.47
26	22.21	6021468.5000	5.79
27	24.62	1742172.7500	1.68
28	31.05	5831833.5000	5.61
29	31.27	5865813.0000	5.64

Figure S2. Positive ESI-LC/MS chromatogram of the ethyl acetate extract

Center for DRUG DISCOVERY RESEARCH and DEVELOPMENT Page 2
Openlynx Report -

Sample: 518 Vial:1:A,8 ID:
File:D21 66 Date:05-Sep-2021 Time:17:18:39
Description:MF1q

Printed: Wed Sep 08 16:32:02 2021

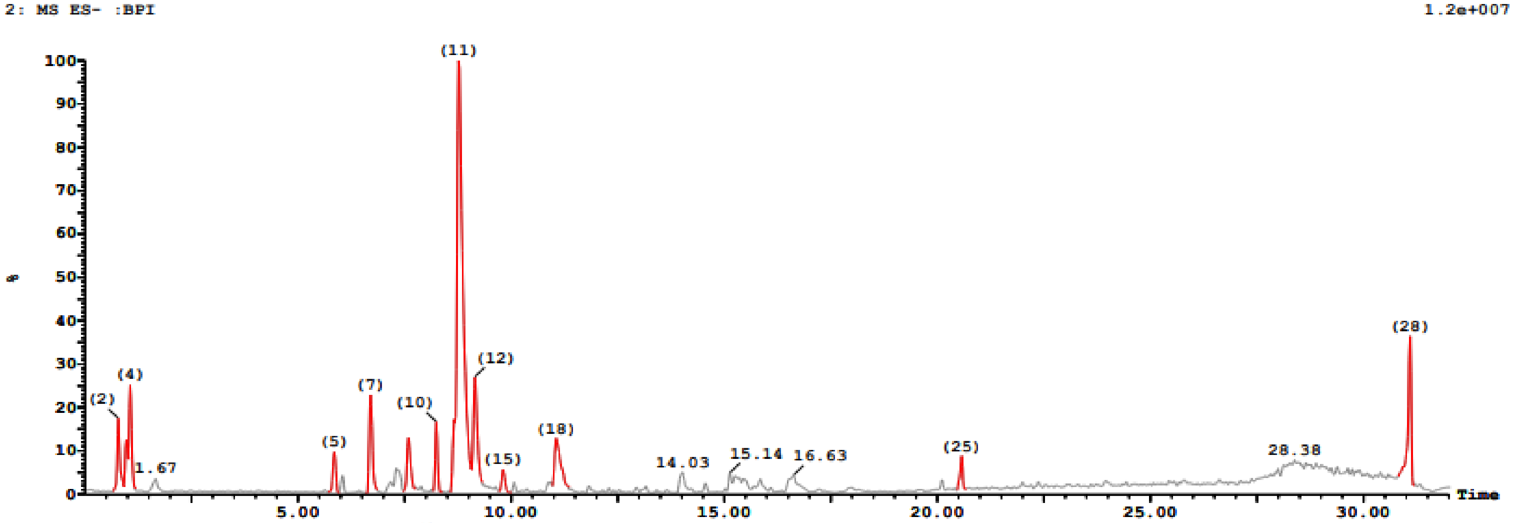

Peak Number	Time	AreaAbs	Area %Total
2	0.78	139378.8125	3.62
3	0.97	75405.1250	1.96
4	1.06	209631.5781	5.45
5	5.85	92627.1953	2.41
7	6.70	238077.5469	6.19
9	7.59	138917.9375	3.61
10	8.25	129174.5391	3.36
11	8.77	1876895.1250	48.79
12	9.15	270406.3750	7.03
15	9.81	53248.4805	1.38
18	11.06	209191.5625	5.44
25	20.57	63722.2227	1.66
28	31.09	350389.1563	9.11

Figure S3. Negative ESI-LC/MS chromatogram of the ethyl acetate extract

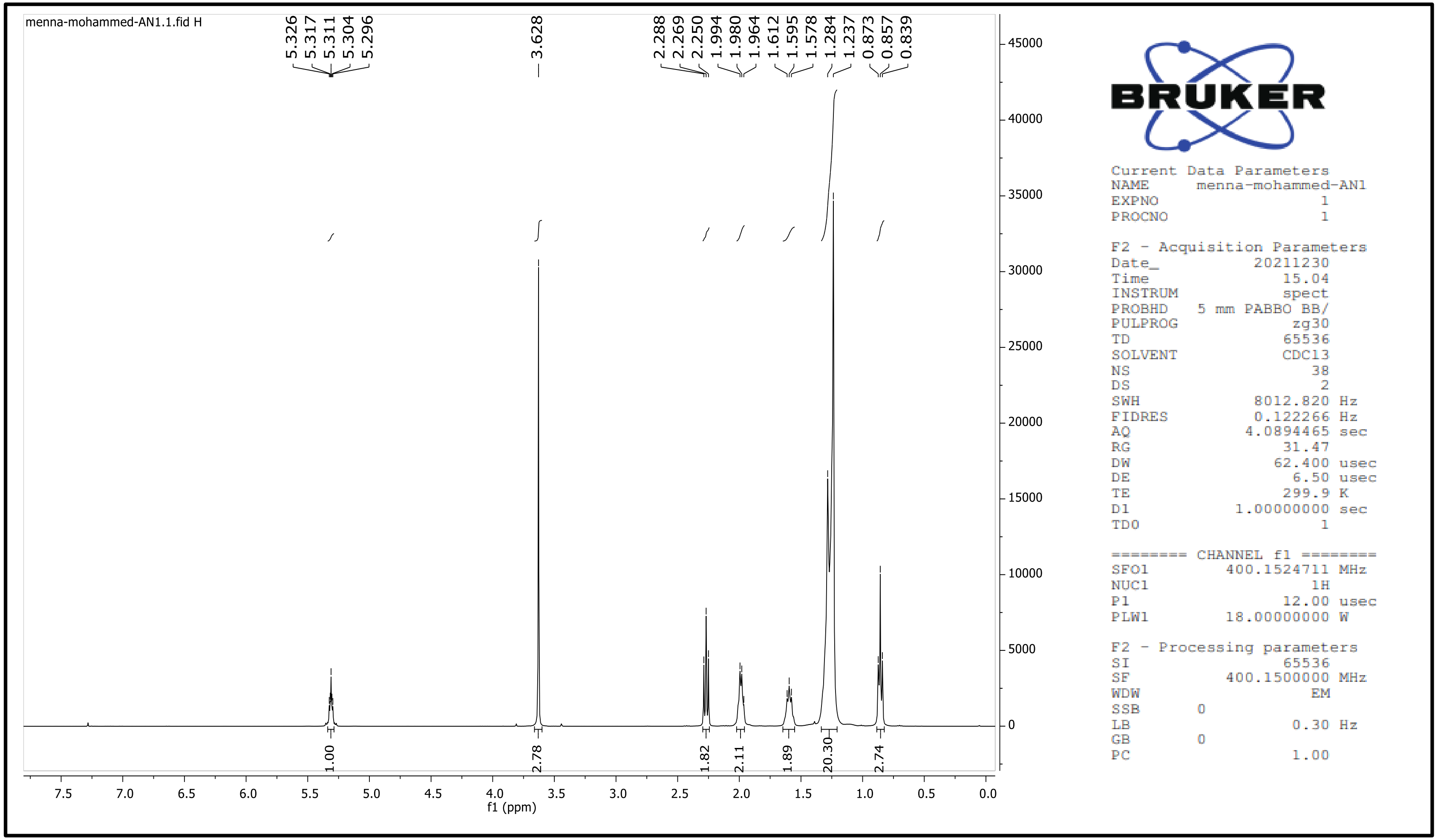

Figure S4. ^{1}H NMR spectrum of compound 1 (CDCL$_3$-*d*, 400 MHz)

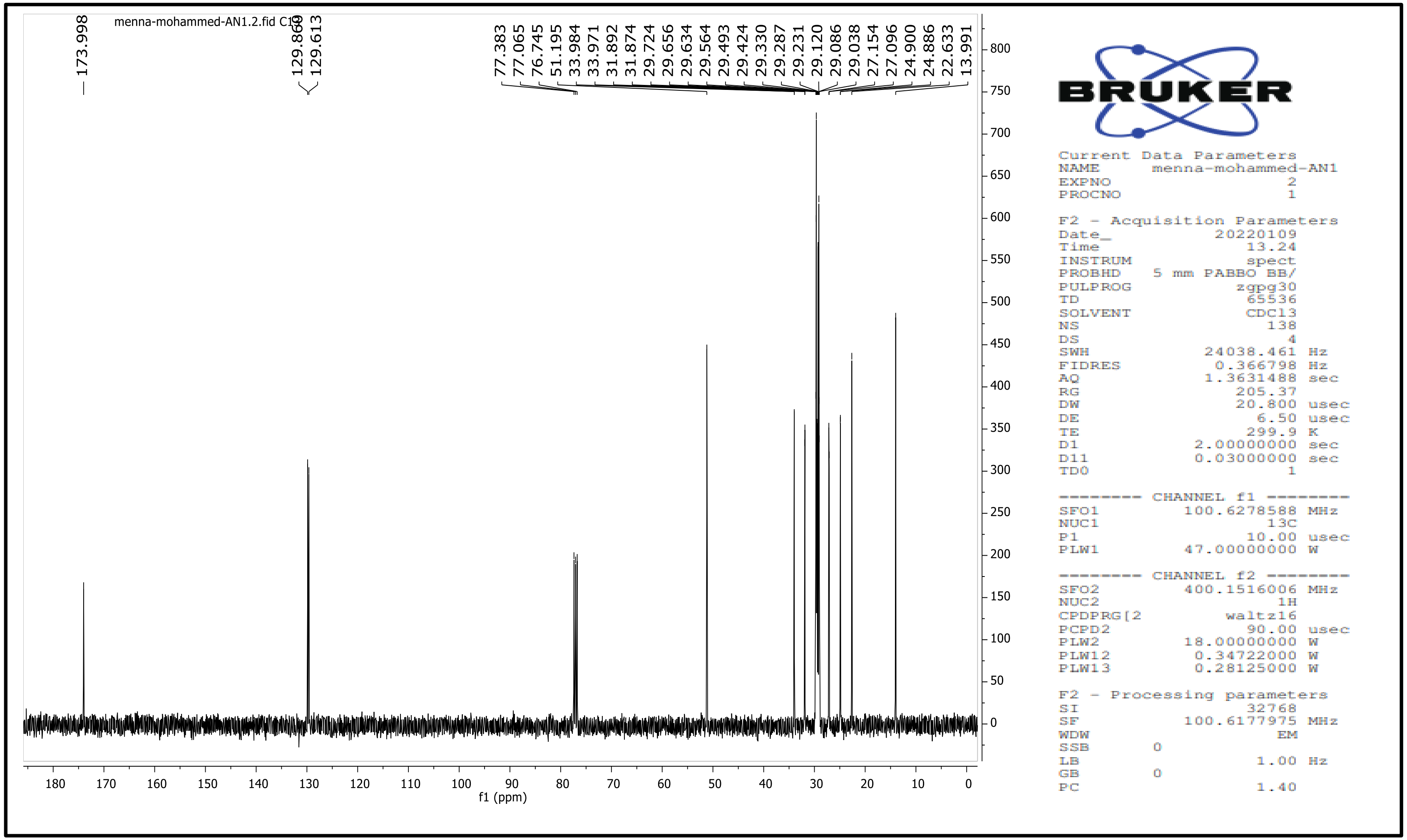

Figure S5. ^{13}C NMR spectrum of compound 1 ($CDCL_3$-*d*, 400 MHz)

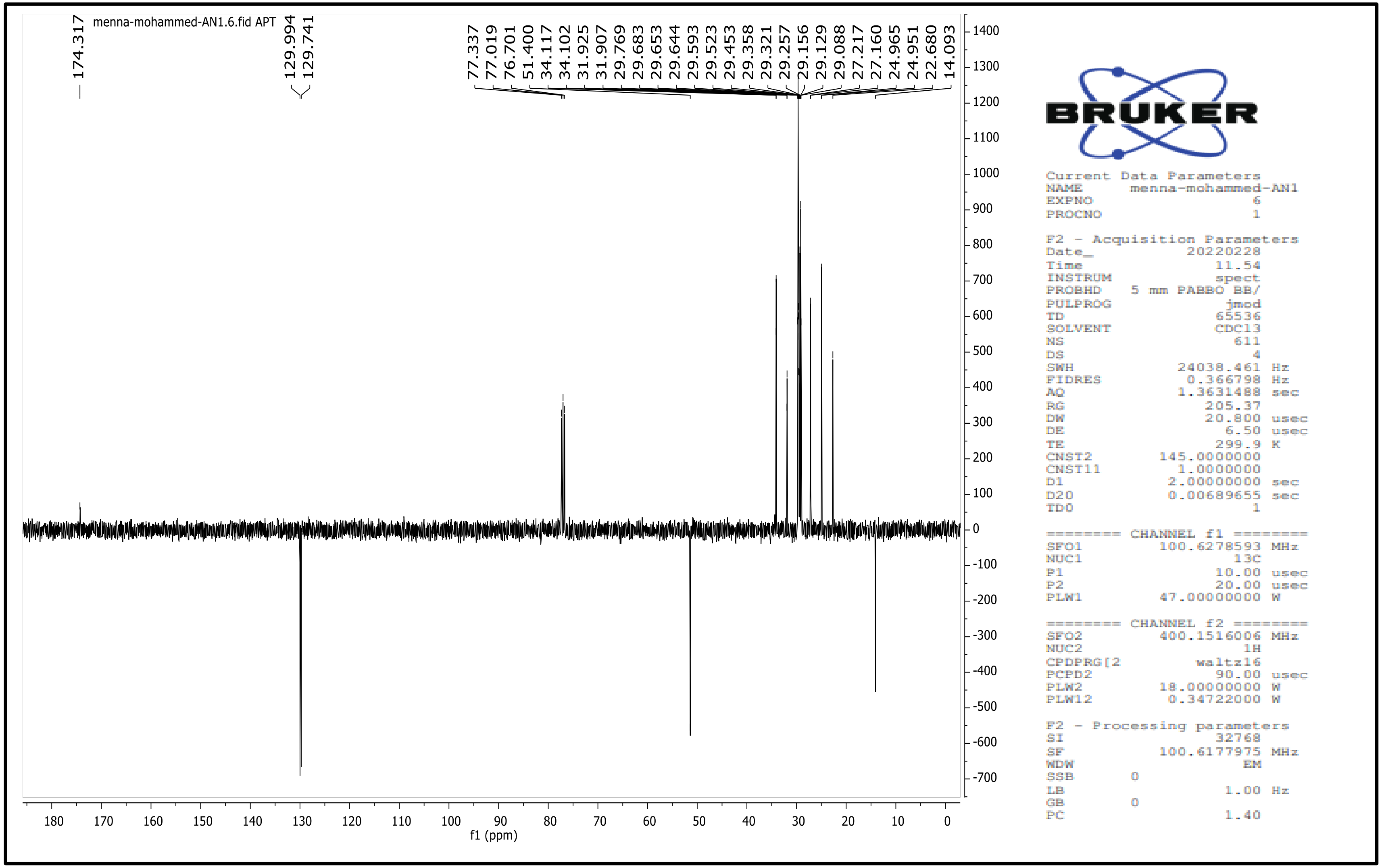

Figure S6. APT spectrum of compound 1 (CDCL$_3$-*d*, 400 MHz)

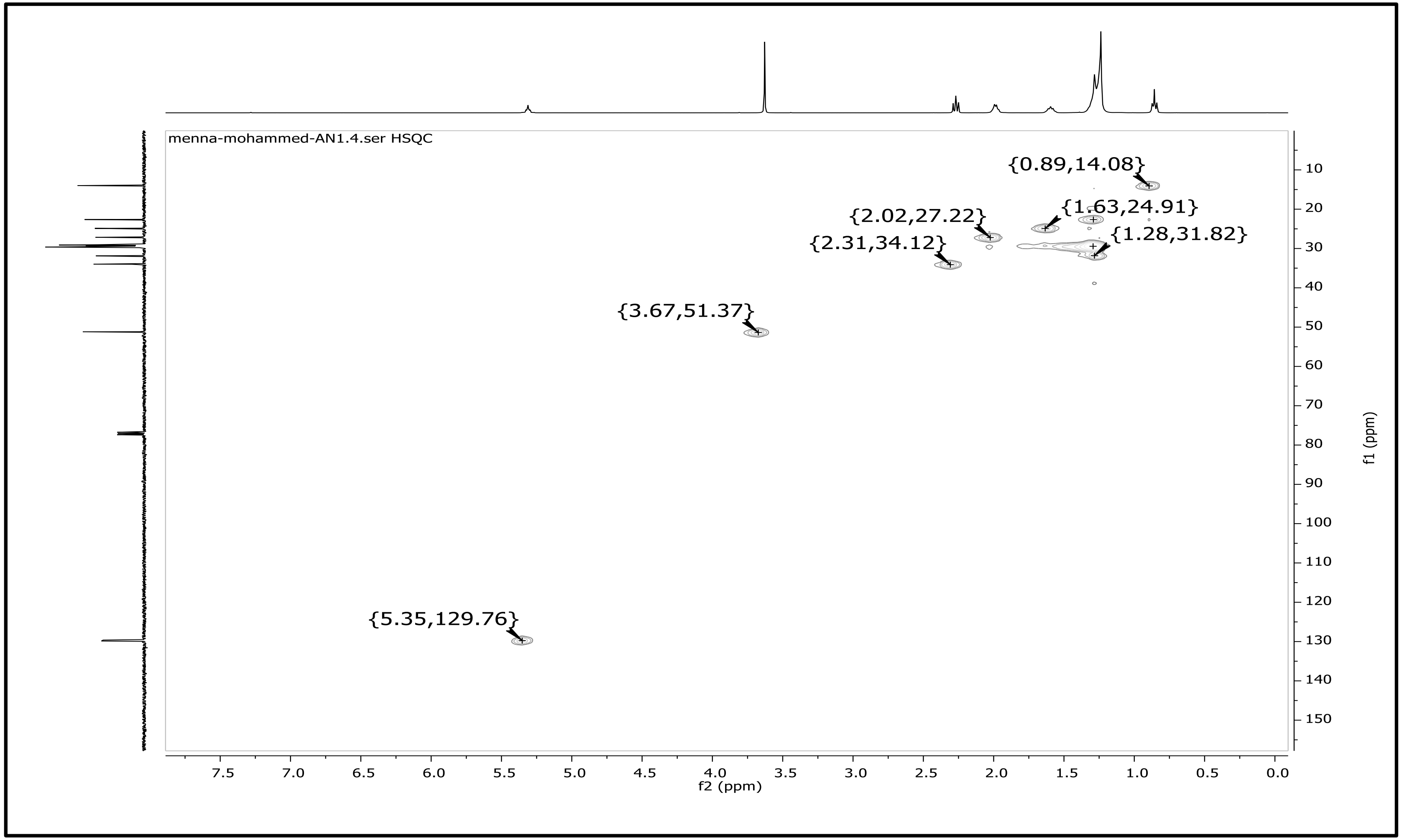

Figure S7. HSQC spectrum of compound 1 ($CDCL_3$-*d*, 400 MHz)

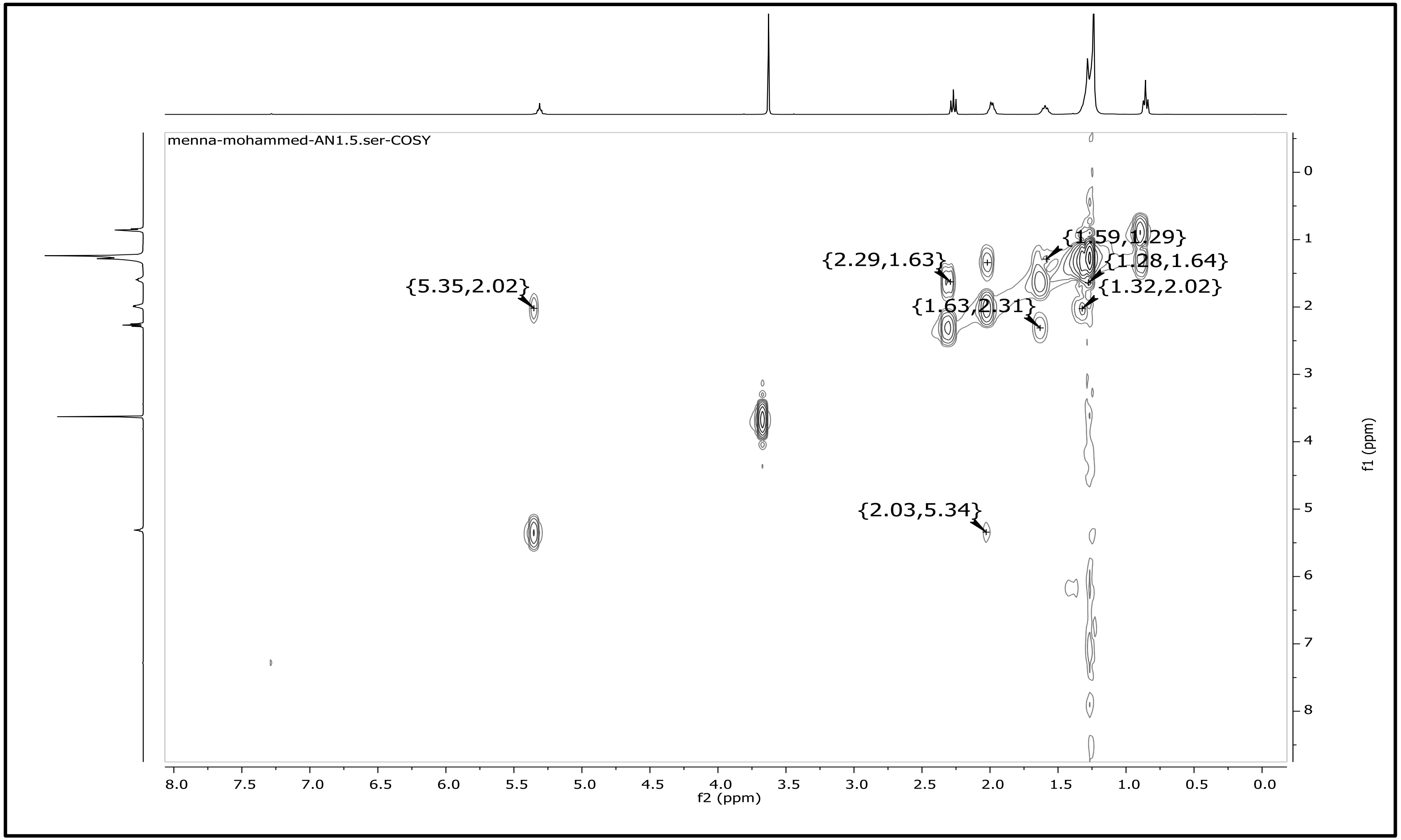

Figure S8. ^{1}H-^{1}H **COSY spectrum of compound 1 ($CDCL_3$-*d*, 400 MHz)**

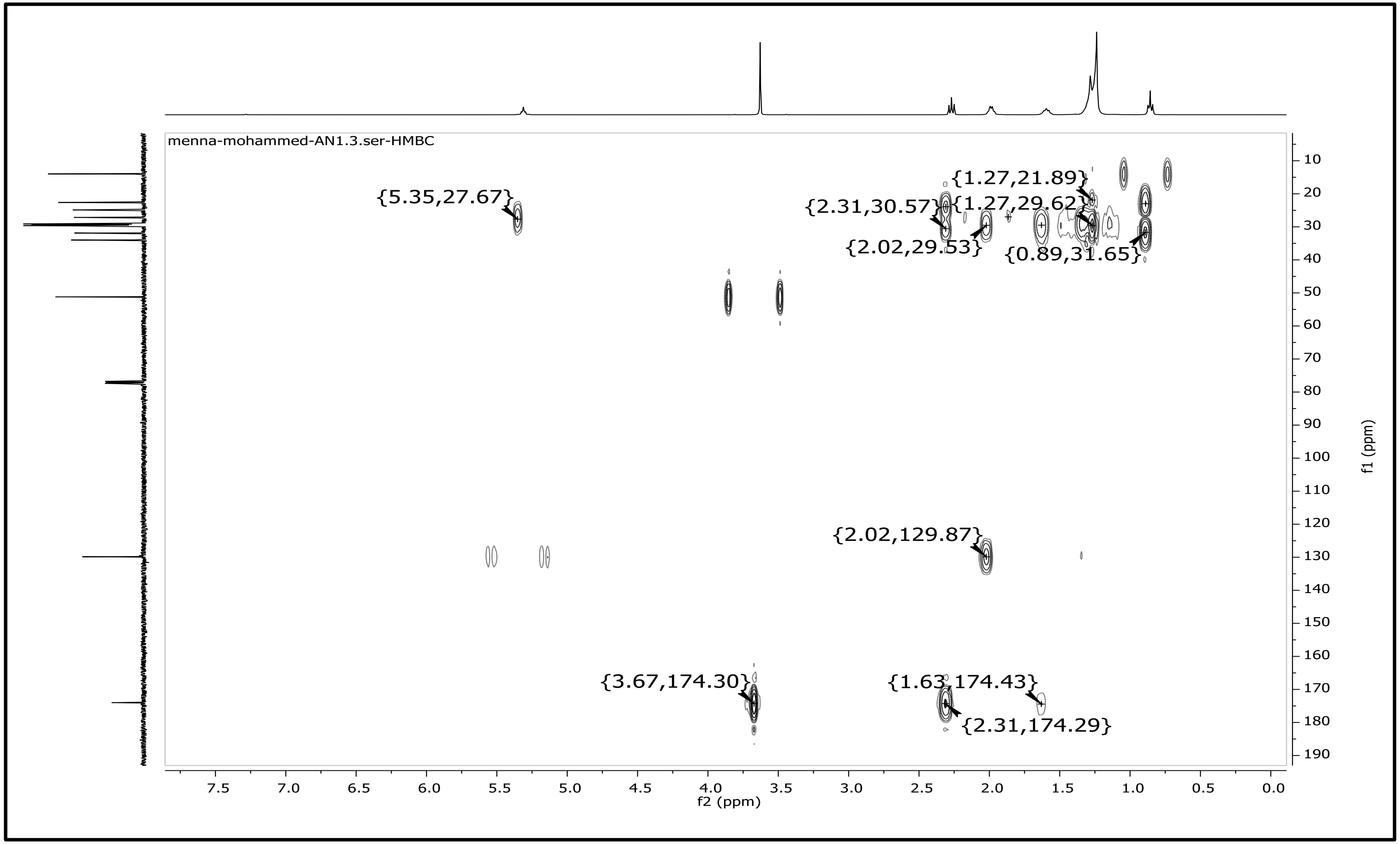

Figure S9. HMBC spectrum of compound 1 (CDCL$_3$-*d*, 400 MHz)

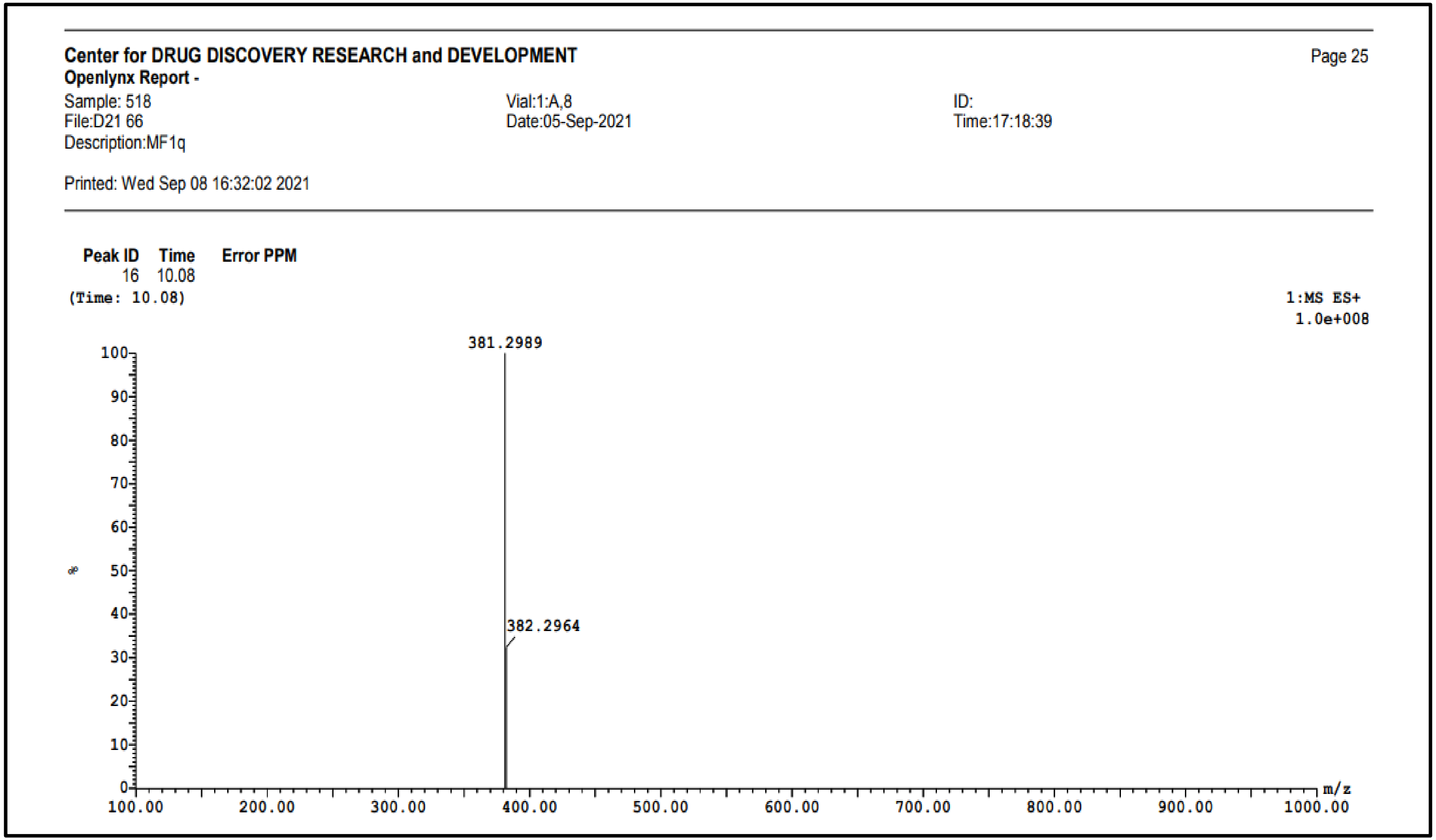

Figure S10. Positive ESI-LC/MS spectrum of compound 1

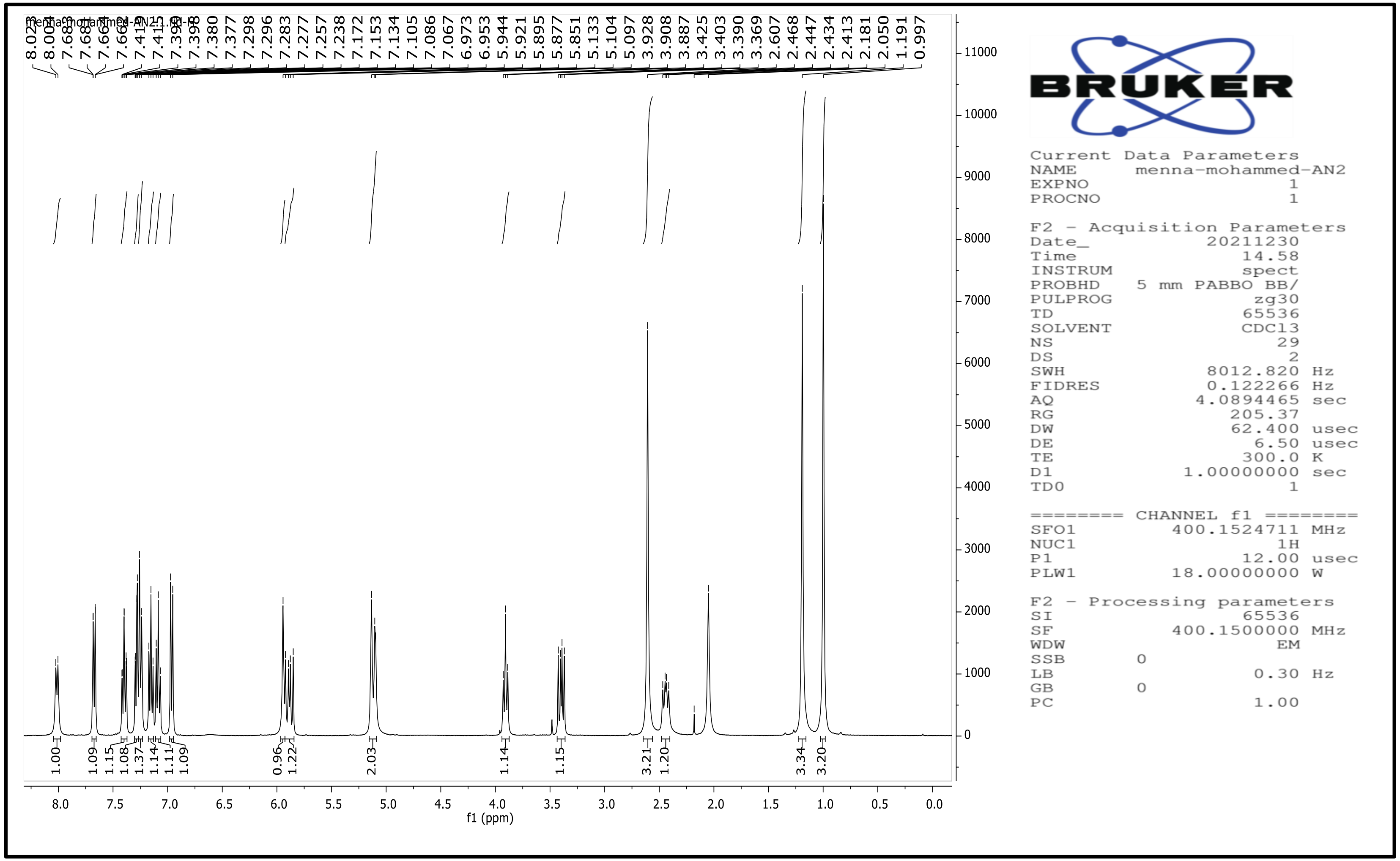

Figure S11. ^{1}H NMR spectrum of compound 2 ($CDCL_3$-*d*, 400 MHz)

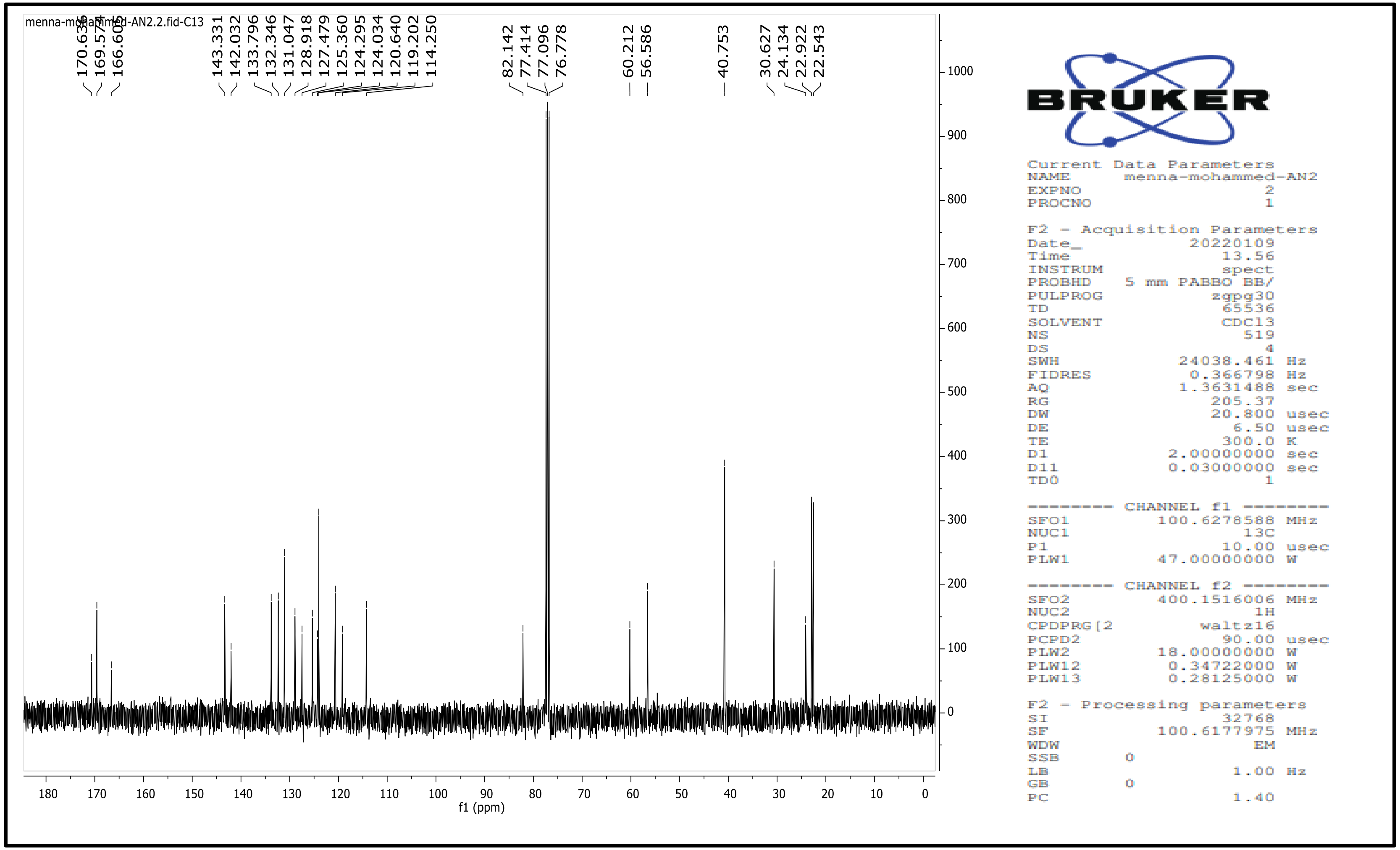

Figure S12. ^{13}**C NMR spectrum of compound 2 (CDCL$_3$-*d*, 400 MHz)**

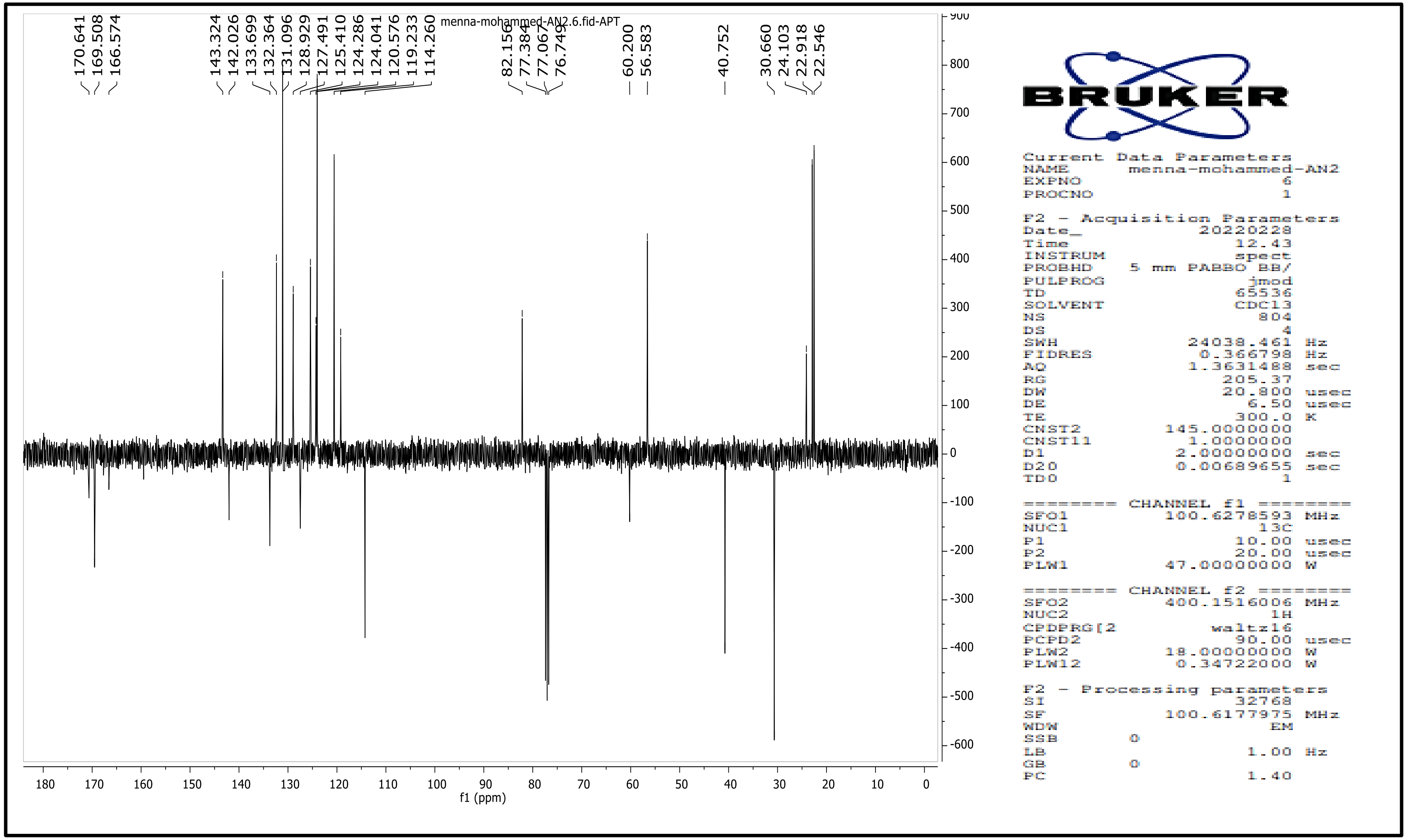

Figure S13. APT spectrum of compound 2 (CDCL3-*d*, 400 MHz)

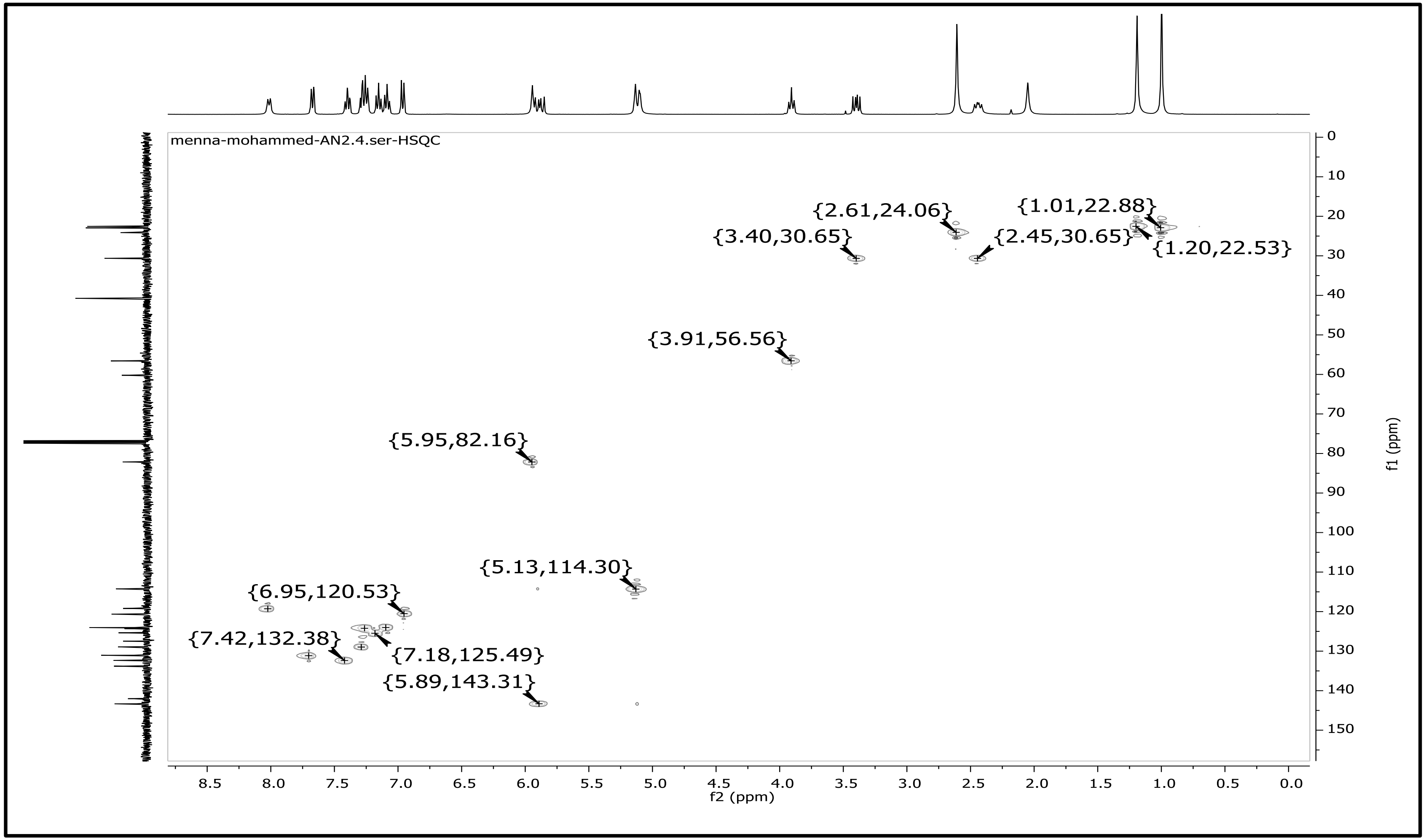

Figure S14. HSQC spectrum of compound 2 ($CDCL_3$-*d*, 400 MHz)

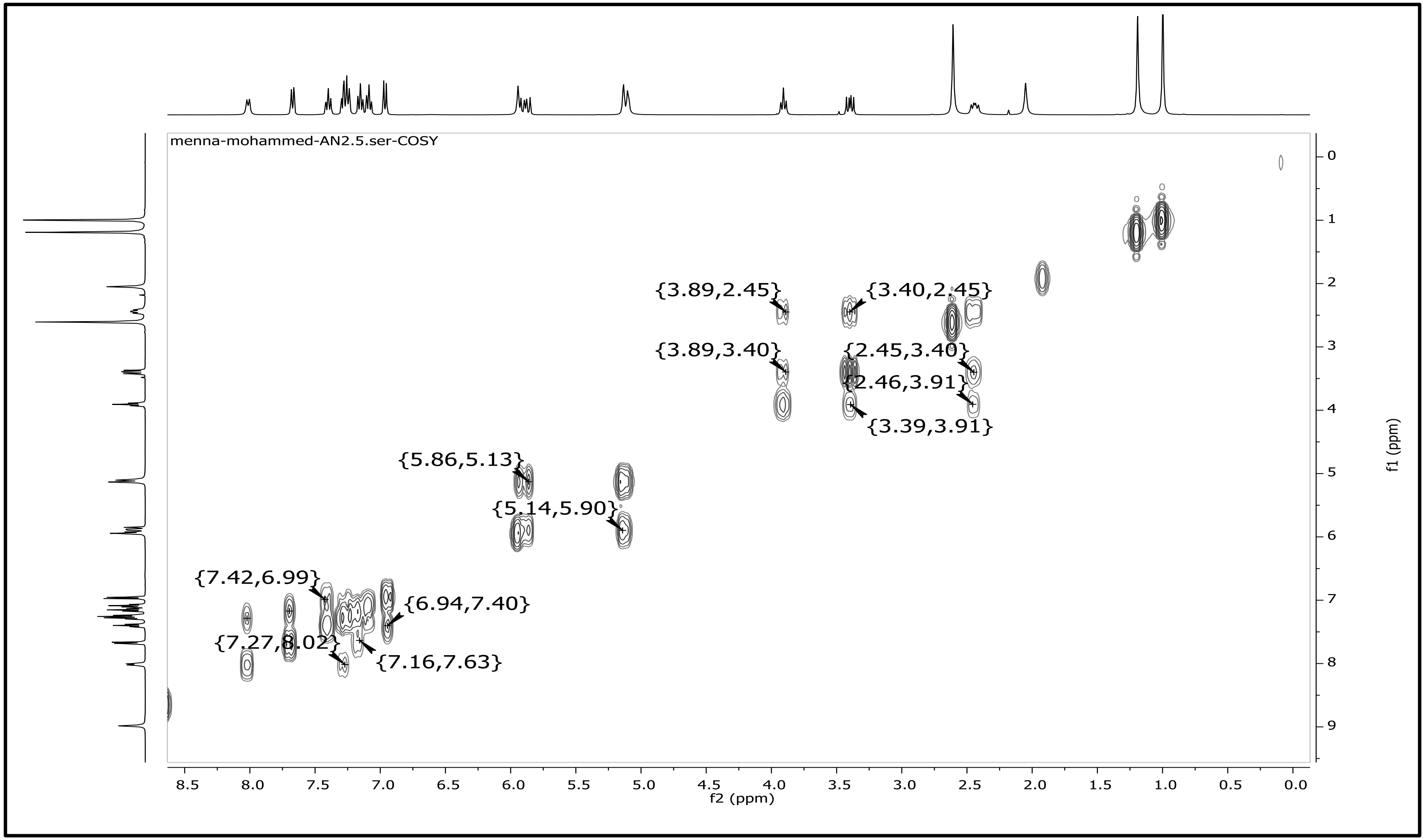

Figure S15. ^{1}H-^{1}H COSY spectrum of compound 2 (CDCL$_3$-*d*, 400 MHz)

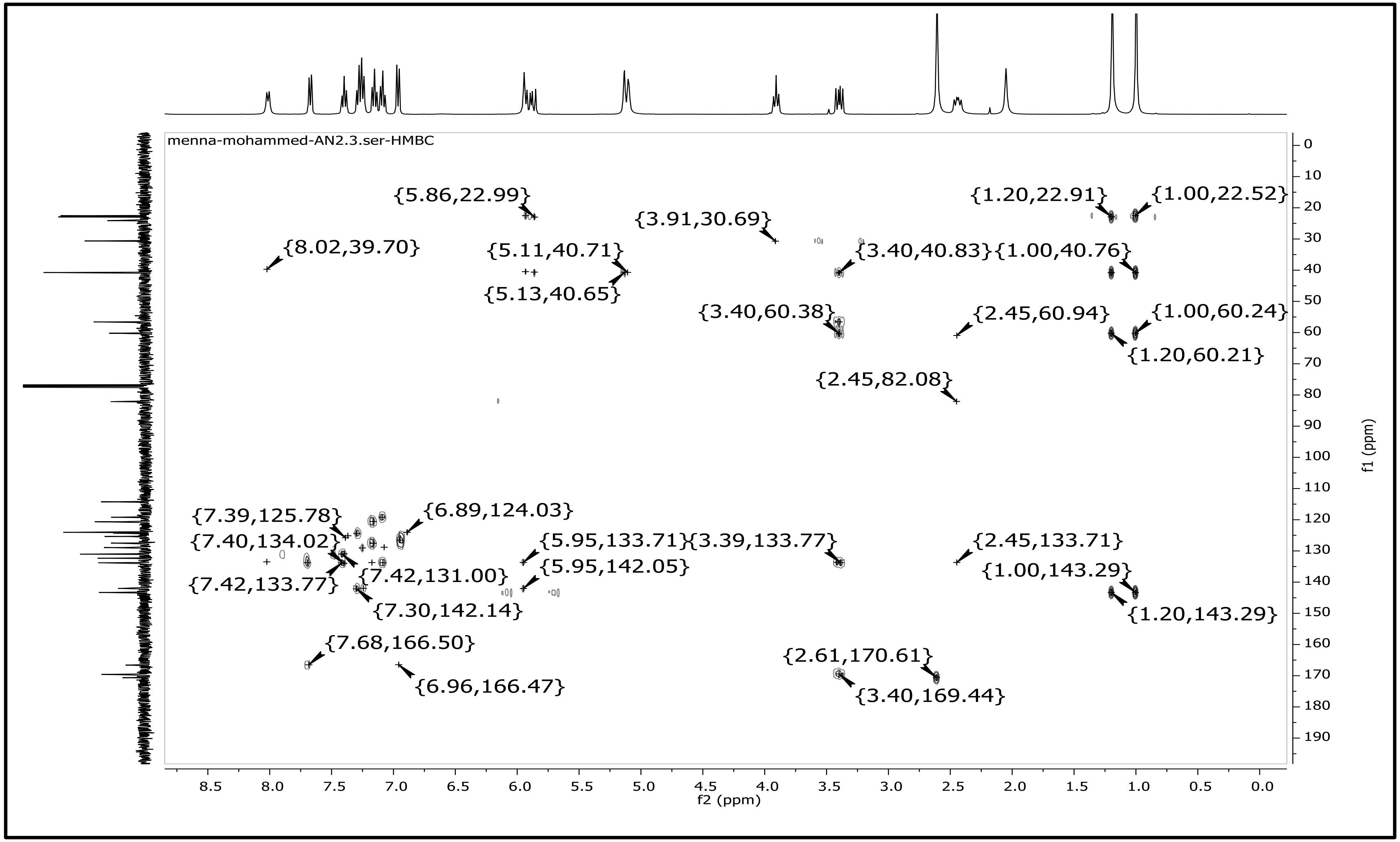

Figure S16. HMBC spectrum of compound 2 (CDCL3-*d*, 400 MHz)

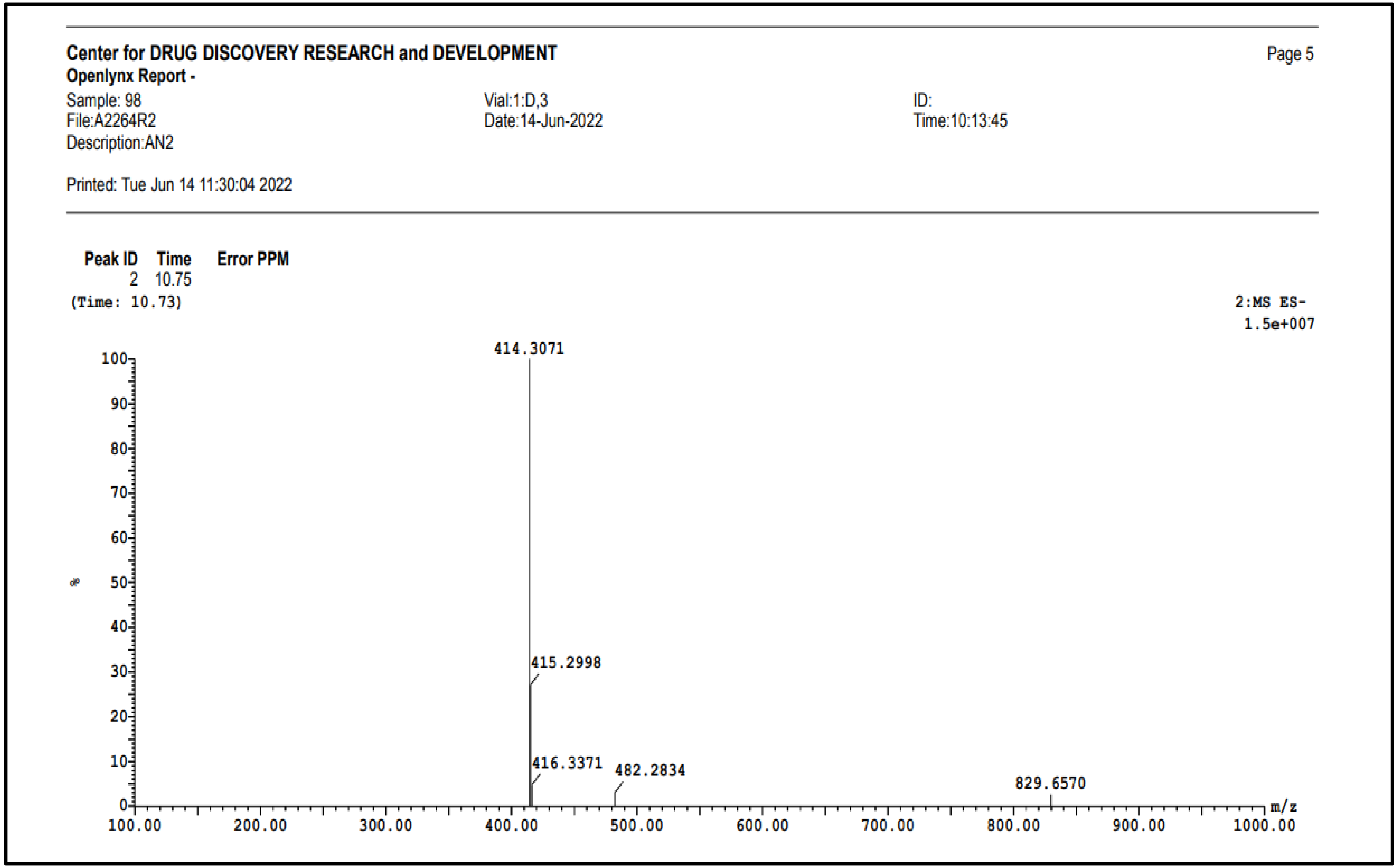

Figure S17. Negative ESI-LC/MS spectrum of compound 2

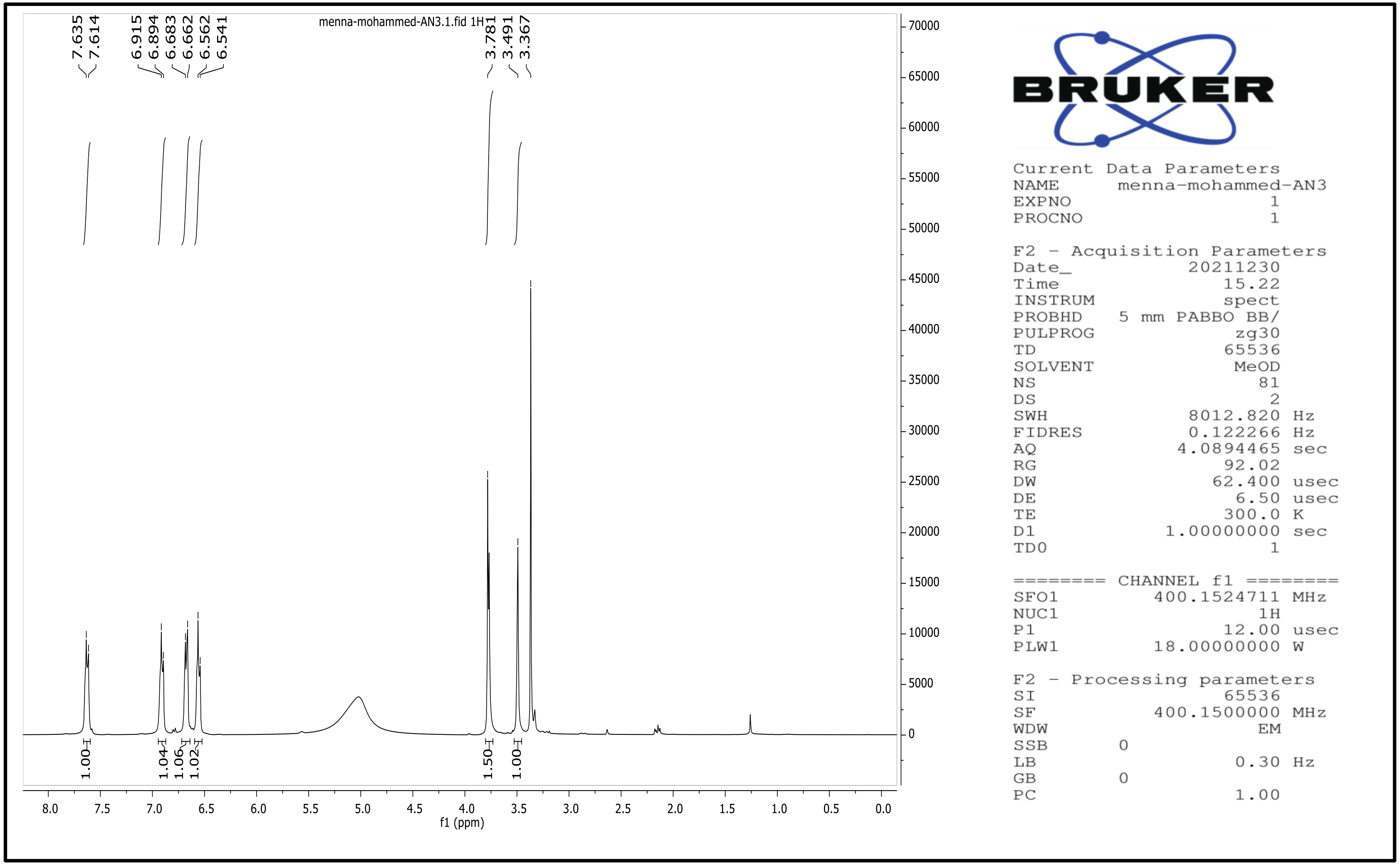

Figure S18. **^{1}H NMR spectrum of compound 3 (CD_3OD-*d*, 400 MHz)**

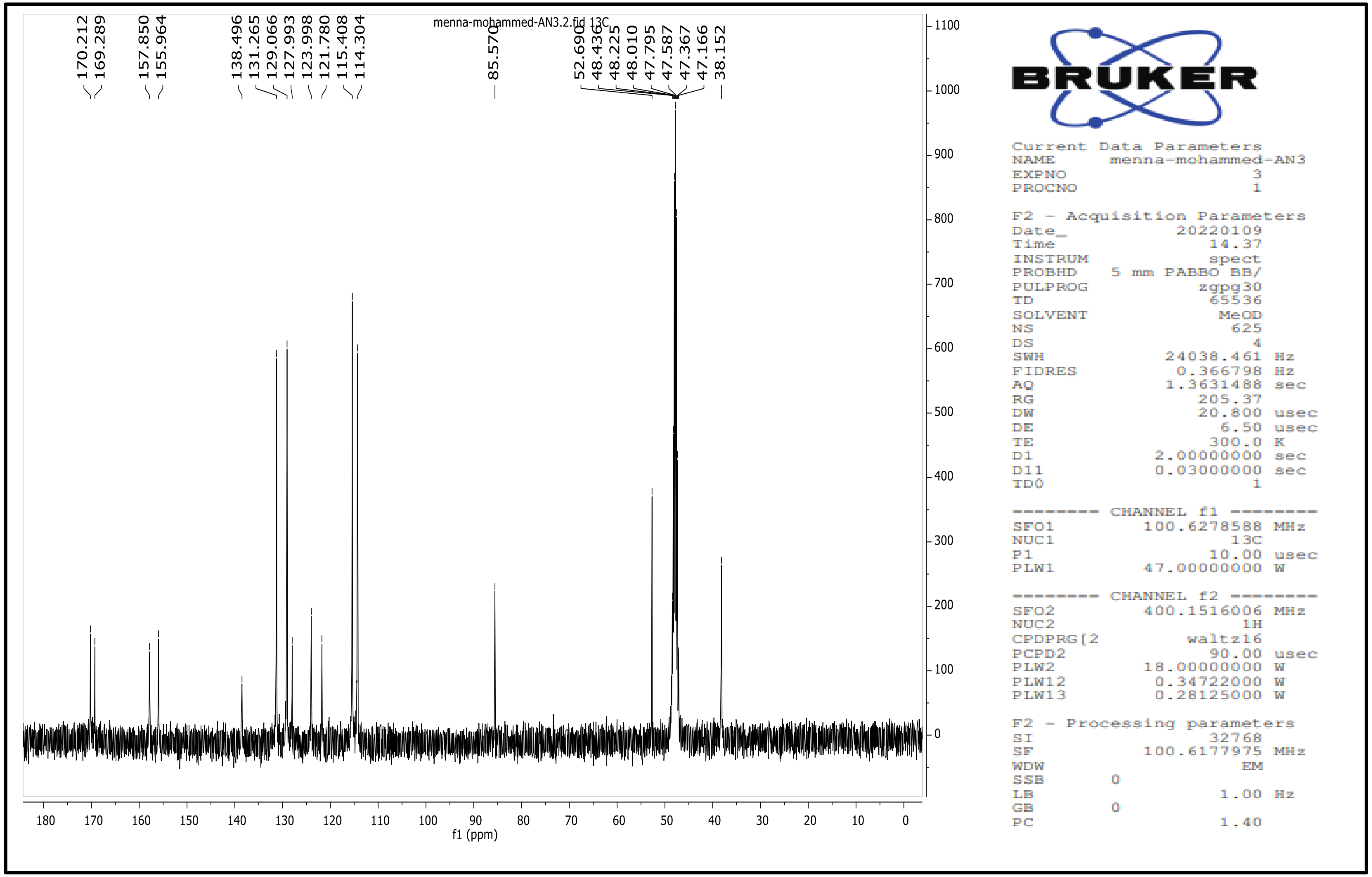

Figure S19. **^{13}C NMR spectrum of compound 3 (CD_3OD-*d*, 400 MHz)**

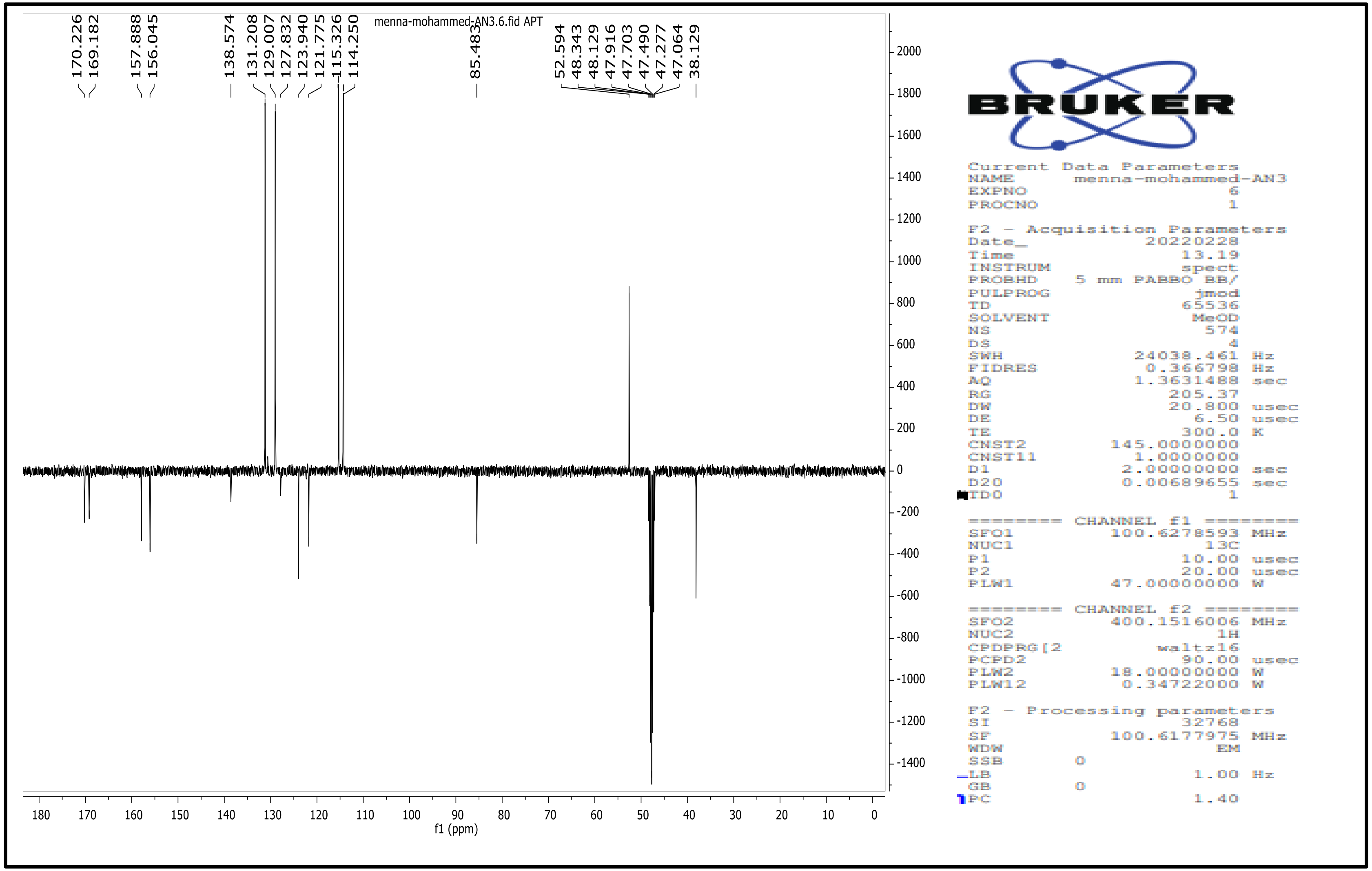

Figure S20. APT spectrum of compound 3 (CD_3OD-*d*, 400 MHz)

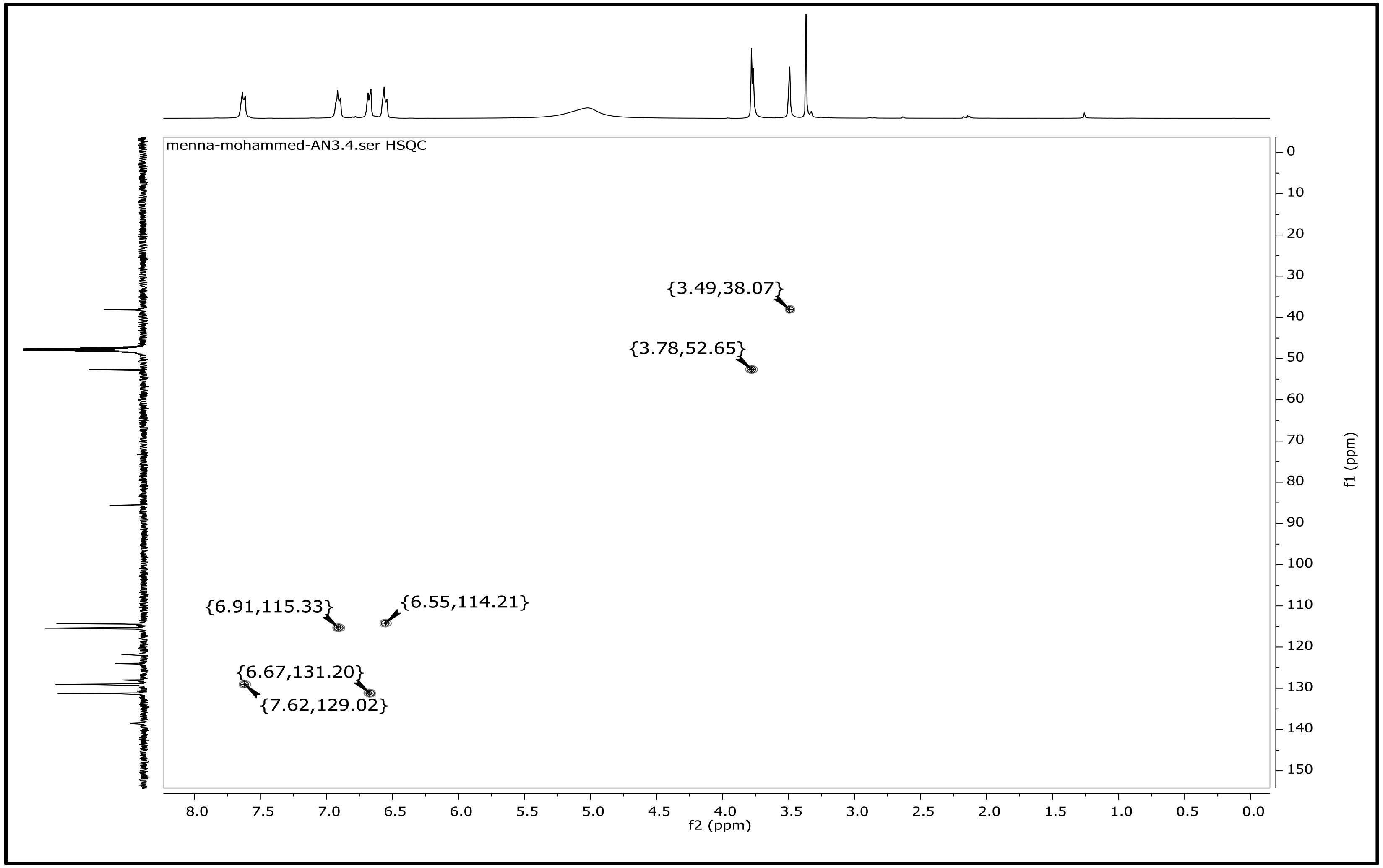

Figure S21. HSQC spectrum of compound 3 (CD_3OD-*d*, 400 MHz)

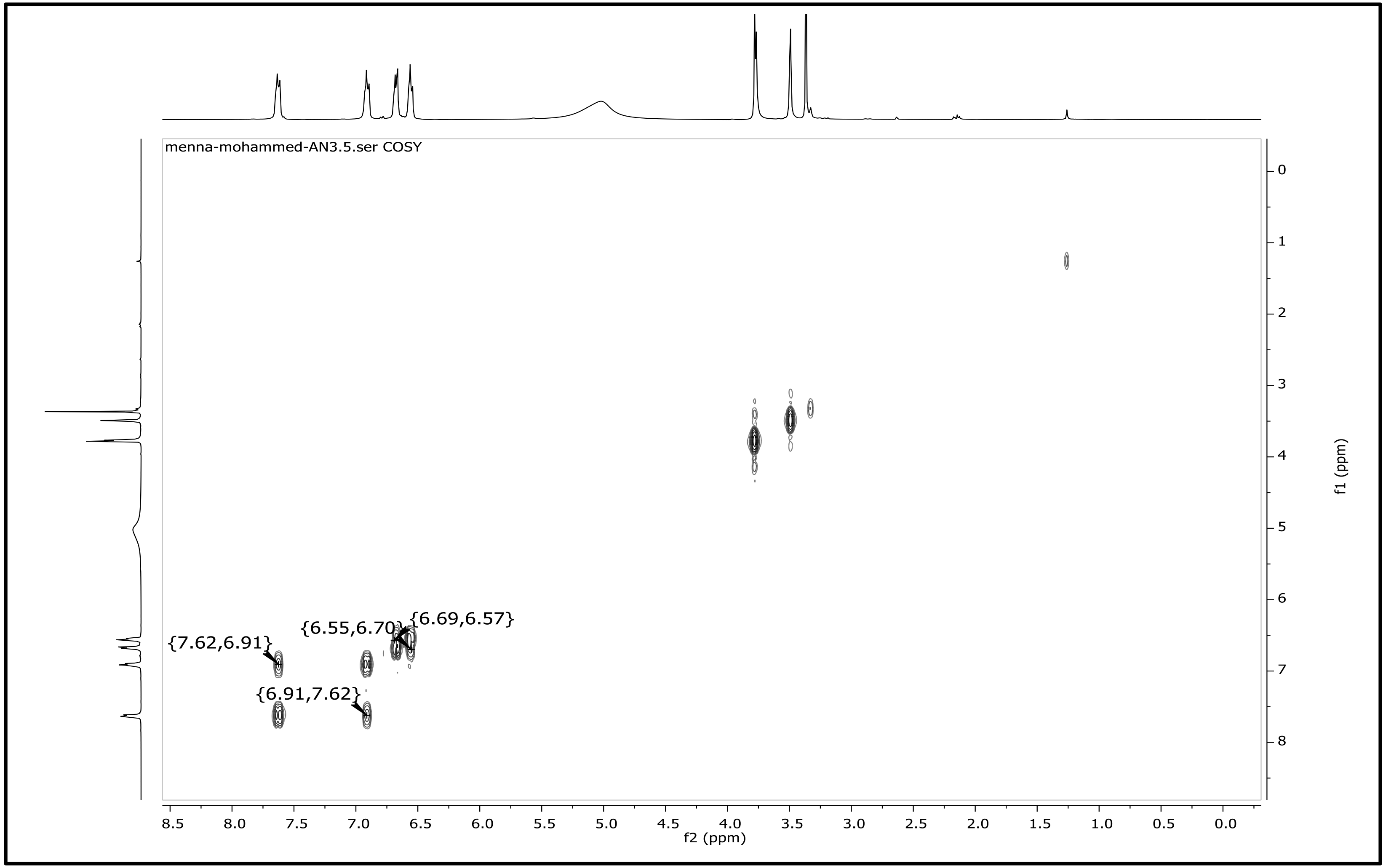

Figure S22. **^{1}H-^{1}H COSY spectrum of compound 3 (CD_3OD-*d*, 400 MHz)**

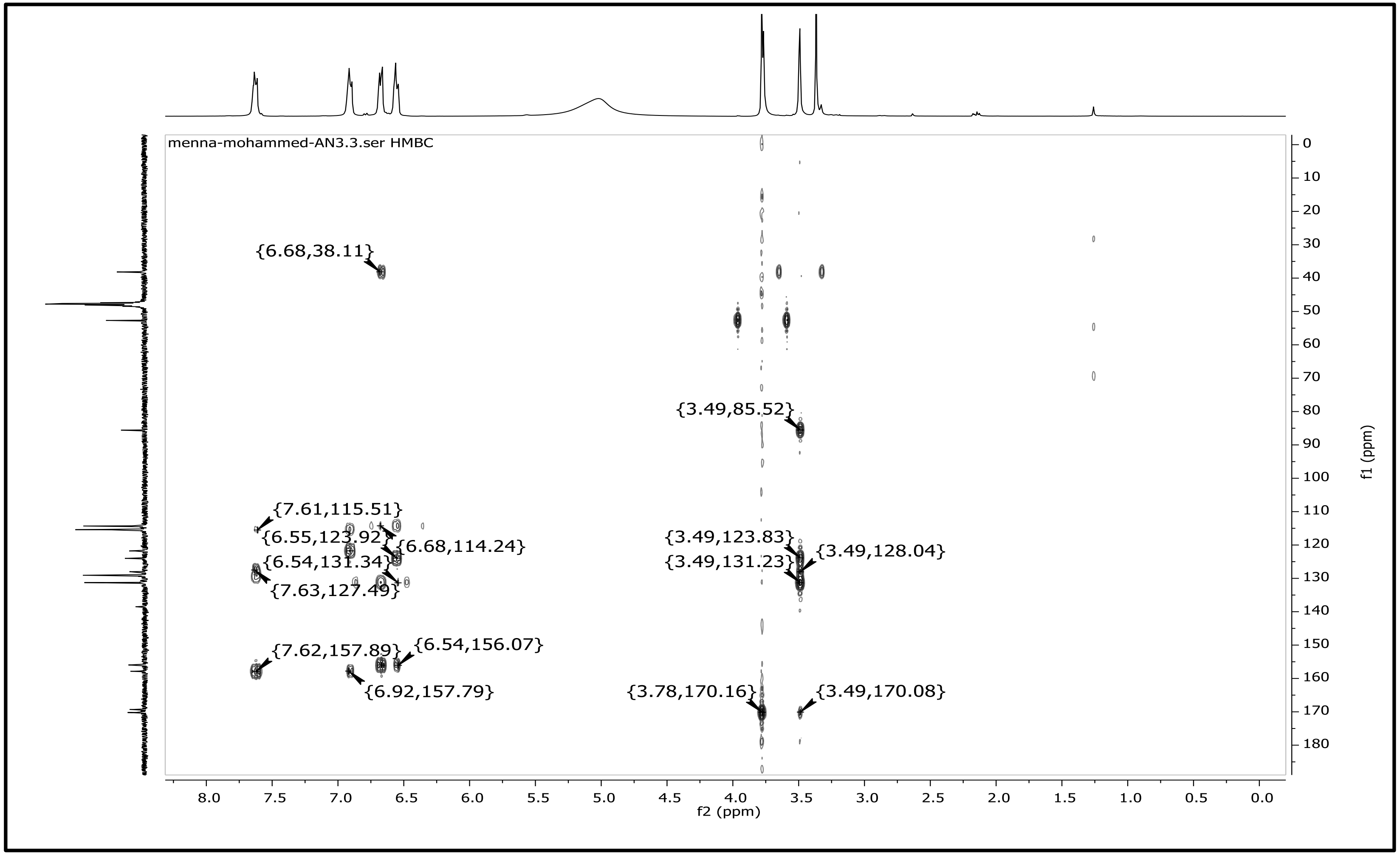

Figure S23. HMBC spectrum of compound 3 (CD_3OD-*d*, 400 MHz)

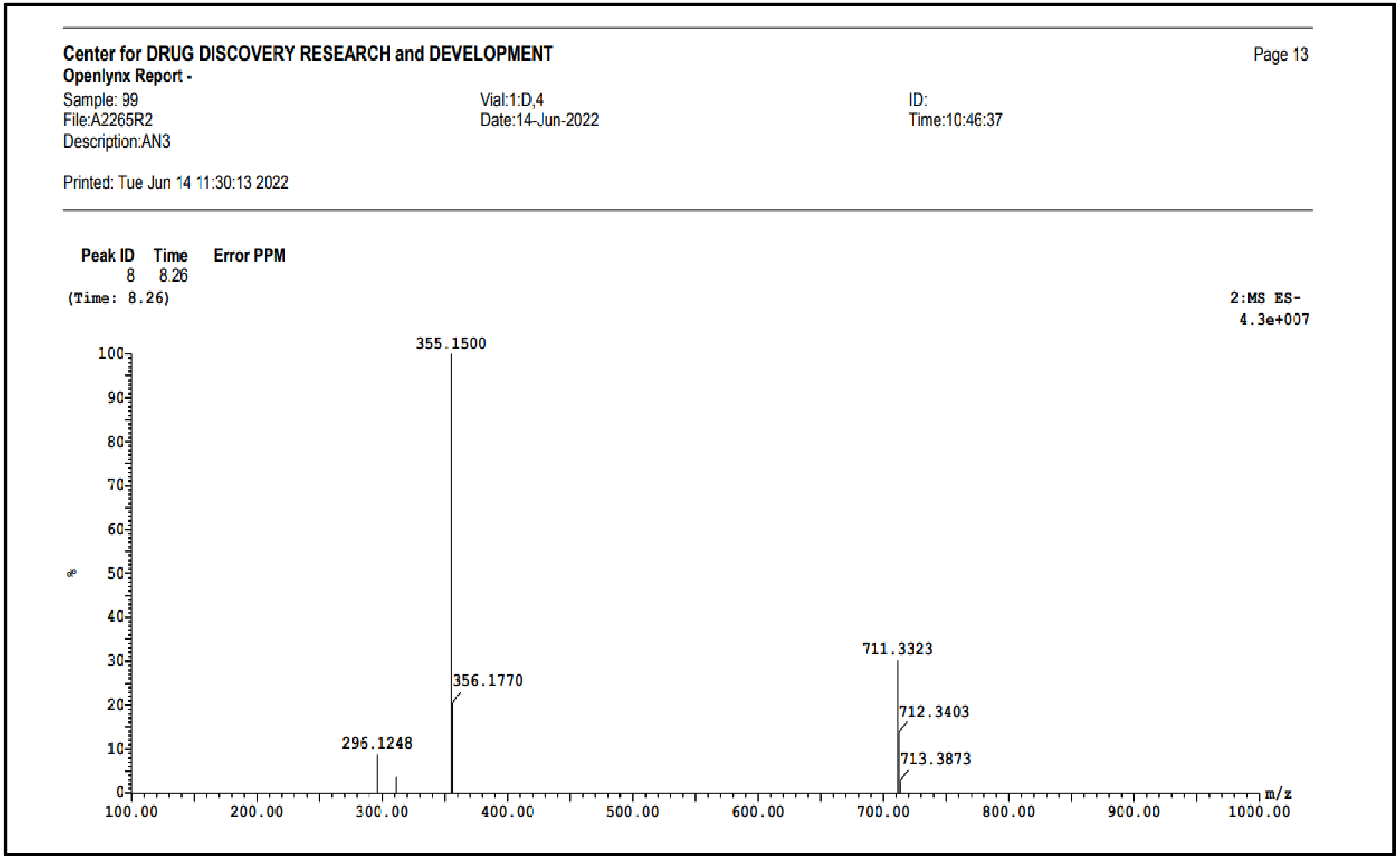

Figure S24. Negative ESI-LC/MS spectrum of compound 3

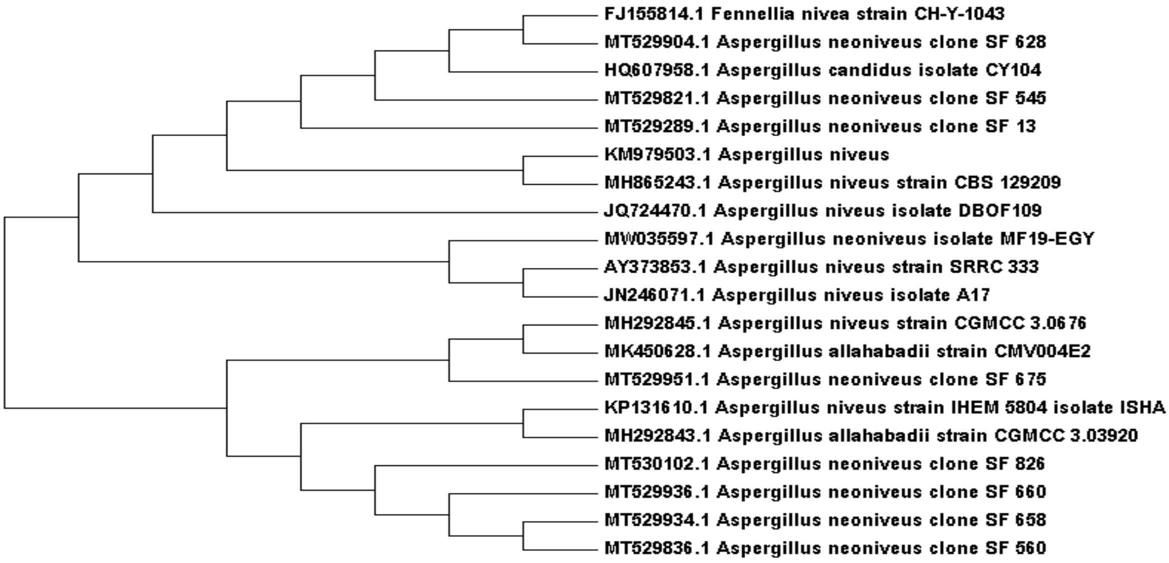

Figure S25. Phylogenetic tree showing relationship of the isolated *Aspergillus neoniveus* with closely related *Aspergillus* species retrieved from GenBank based on their sequence homologies of 18srDNA.

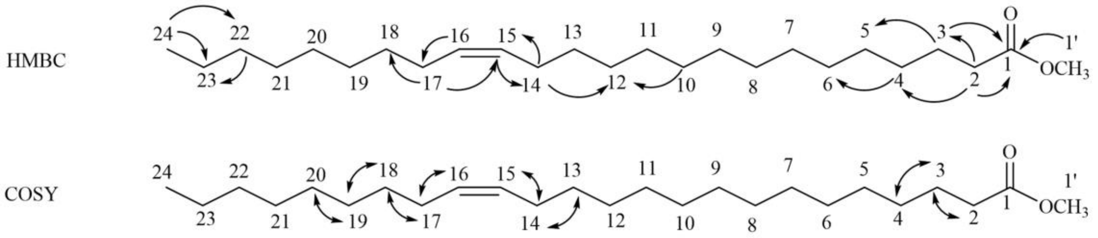

Compound 1

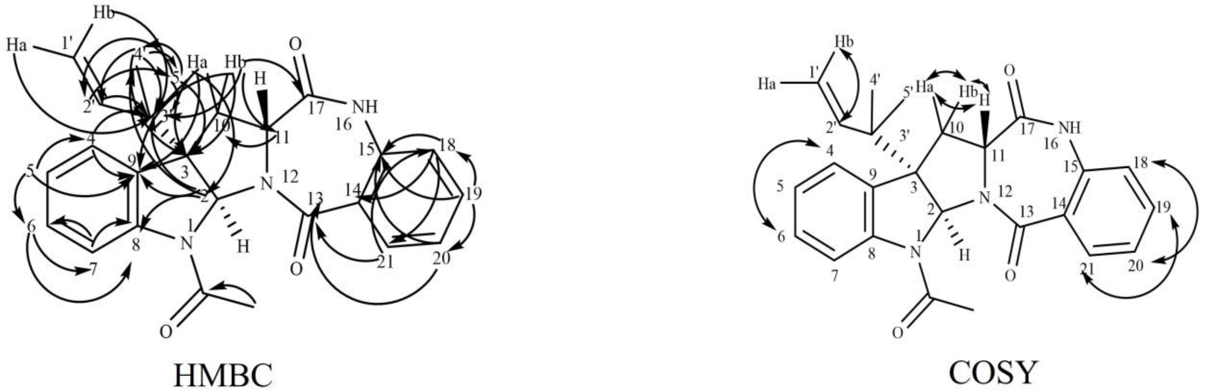

Compound 2

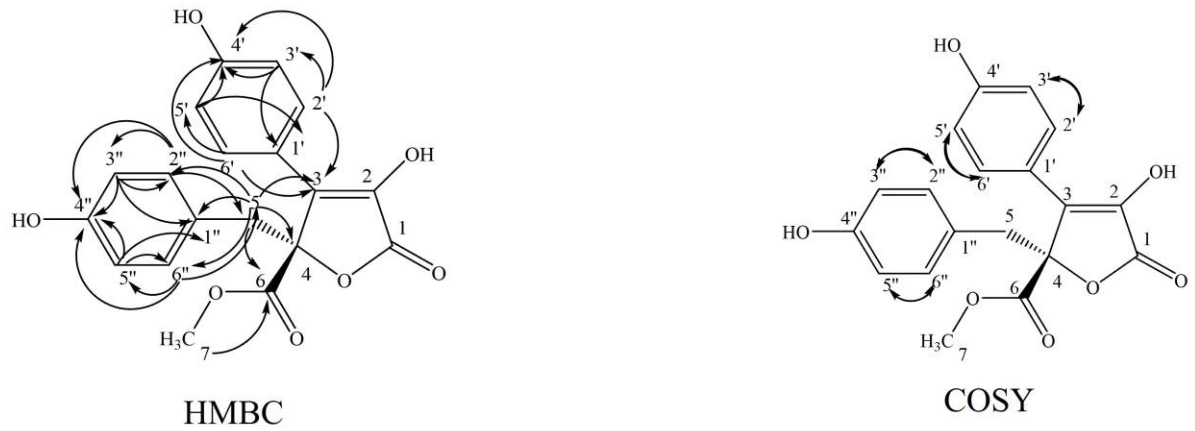

Compound 3

Figure S 26. Schematic representation of HMBC and COSY correlations in compounds 1-3
